# Autism Spectrum Disorder: Integrating Genetic and Environmental Risk

**DOI:** 10.3390/cells15110985

**Published:** 2026-05-27

**Authors:** Satoshi Kamijo, Hideki Miwa, Kazutaka Ikeda

**Affiliations:** 1 Department of Neuropsychopharmacology, National Institute of Mental Health, National Center of Neurology and Psychiatry, Kodaira 187-8553, Tokyo, Japan; kamijo@ndmc.ac.jp (S.K.);; 2 Department of Physiology, National Defense Medical College, Tokorozawa 359-8513, Saitama, Japan; 3 Tokyo Metropolitan Institute of Medical Science, Setagaya 156-8506, Tokyo, Japan

**Keywords:** autism spectrum disorder (ASD), genetic factors, maternal immune activation, excitation/inhibition imbalance, GABAergic system, brain–gut interaction, serotonin, oxytocin, dopamine, endocannabinoid system, cerebellum

## Abstract

**Highlights:**

**What are the main findings?**

- Genetic Architecture: Both de novo and inherited genetic factors contribute to ASD development.
- Environmental Impact: Environmental factors generally provide a modest increase in ASD risk.

**What are the implications of the main findings?**

- Biological Convergence: Genetic and environmental risk factors converge on specific biological pathways during critical developmental windows.
- Gene × Environment Significance: The variable susceptibility of environmental risks underscores the importance of gene × environment interactions.

**Abstract:**

Autism spectrum disorder is a common neurodevelopmental condition, defined by persistent deficits in social interaction and communication, as well as restricted repetitive patterns of behavior, interests, or activities. Autism spectrum disorder is highly heterogeneous, encompassing a broad range of clinical presentations and suggesting it includes multiple etiological subtypes. Although no unified cause has been established, accumulating evidence indicates that genetic susceptibility interacts with environmental and developmental factors to shape diverse phenotypic outcomes. This review summarizes epidemiological findings and discusses major proposed etiological mechanisms, integrating evidence from human studies and animal models. Although animal models are not directly translatable to humans, their findings provide mechanistic insights that bridge epidemiological observations with neurobiological hypotheses.

## 1. Introduction

Autism spectrum disorder (ASD, also known as autism spectrum condition) is a common neurodevelopmental condition. The fifth edition of the Diagnostic and Statistical Manual of Mental Disorders (DSM-5) defines ASD as a condition characterized by the following two criteria: (A) Persistent deficits in social communication and social interaction across multiple contexts, and (B) Restricted, repetitive patterns of behavior, interests, or activities. In addition to these core behavioral characteristics, individuals with ASD often exhibit accompanying comorbidities such as attention-deficit/hyperactivity disorder, anxiety disorder, intellectual disability, epilepsy, sleep disorder, and hyper/hyposensitivity [1]. Together with the core symptoms, these comorbid conditions contribute to increased functional difficulties in individuals with ASD. Despite substantial research efforts, the etiology of ASD remains elusive.

This study was conducted as a narrative review of the literature. Relevant studies were identified through searches of PubMed and Google Scholar, focusing on key and peer-reviewed publications addressing the etiology of ASD. Search terms included combinations of “autism spectrum disorder”, “genetics”, “environmental factors”, “risk”, “epidemiological”, “etiology” along with related terms. Additional searches were performed using section-specific keywords. Although priority was given to recent studies, earlier landmark studies were also included when relevant. In addition, reference lists of relevant review articles were screened to identify foundational studies in epidemiology, human research, and animal models.

To minimize the risk of overlooking influential studies, supplementary searches were conducted to identify highly cited publications in the field, with assistance from AI-based tools such as ChatGPT and Gemini. AI-based tools were used only to assist in identifying potentially relevant publications. All AI-suggested references were manually evaluated for relevance and accuracy prior to inclusion. Almost all papers suggested by AI-based tools were already present in our preliminary manually curated reference list, indicating that these tools played only a supplementary role in the review process.

Although numerous etiological hypotheses for ASD have been proposed, we focused on those supported by evidence from human studies, excluding hypotheses with limited or no human-based empirical support. Particularly for environmental risk factors, where numerous hypotheses have been proposed, priority was given to those supported by reproducible epidemiological findings. For associations with more limited reproducibility, greater emphasis was placed on hypotheses additionally supported by complementary evidence from human anatomical or functional studies, or animal experiments. Methodological rigor and influence in the field were also taken into consideration.

For evidence synthesis, the collected evidence was qualitatively integrated and compared across studies. When conflicting findings existed, we explicitly discussed such discrepancies and placed greater emphasis on methodologically robust and reproducible findings. Particular weight was given to human epidemiological evidence, as direct interventional studies on ASD etiology are generally not feasible.

**Figure 1:**
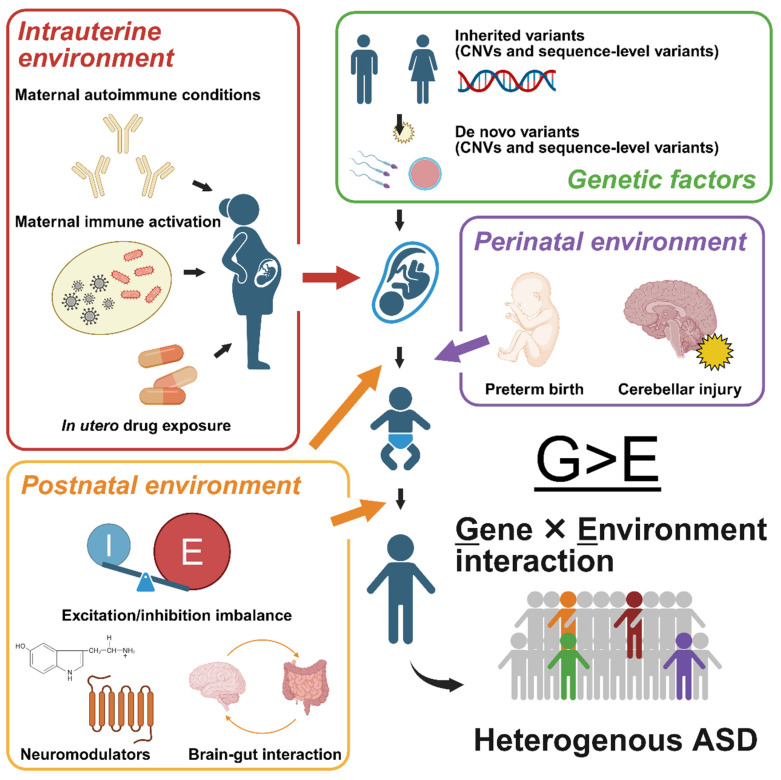
Conceptual framework of candidate etiological factors. Factors that are discussed in this review are organized along the child’s developmental trajectory. ASD, autism spectrum disorder; CNVs, copy number variants; G, gene; E, environment. Created in BioRender. Kamijo, S. (2026) https://BioRender.com/0s8w0p9 (accessed on 25 May 2026).

The aim of this review is to provide an overview of the diverse risk factors proposed for ASD and to critically compare these hypotheses within a unified framework (Figure 1), with the goal of clarifying their relative contributions and the strength of supporting evidence.

## 2. Epidemiology of ASD

The estimated prevalence of autism spectrum disorder (ASD) varies substantially across studies because of methodological differences, including study period, region, age group, case definition, and data source [2,3,4]. A meta-analysis of 79 studies from 2000 to 2020 reported a pooled global prevalence of 0.72% (95% confidence interval = 0.61–0.85) [5]. Another study estimated the prevalence to be 0.79% or approximately 61.8 million people worldwide [6]. The latter study ranked ASD as the leading cause of non-fatal health burden in individuals under 20 years of age.

In the United States, the Autism and Developmental Disabilities Monitoring Network has tracked ASD prevalence among 8-year-old children biennially since 2000 [7]. During this time, the prevalence increased from approximately 1 in 150 children in 2000 to 1 in 68 in 2010 and 1 in 31 in 2022. Autism spectrum disorder has consistently been more common in boys than in girls, with a male-to-female ratio of approximately 3.4:1, although the observed male-biased prevalence may result from biases in diagnostic tools, particularly their reduced sensitivity in identifying ASD in females [8]. Prevalence estimates vary depending on the data source. In 2022, US estimates were 19.1, 20.2, and 33.2 per 1000 based on special education records, Medicaid claims, and the National Survey of Children’s Health, respectively. Despite variations, prevalence in high-income countries remains within a similar range.

The prevalence of ASD has increased over time, largely because of changes in diagnostic criteria, better detection, and social and policy factors, such as greater awareness and the use of an ASD diagnosis to access educational and clinical support.

## 3. Genetic Cause of ASD

### 3.1. Family Based Evidence for Genetic Architecture in ASD

**Figure 2:**
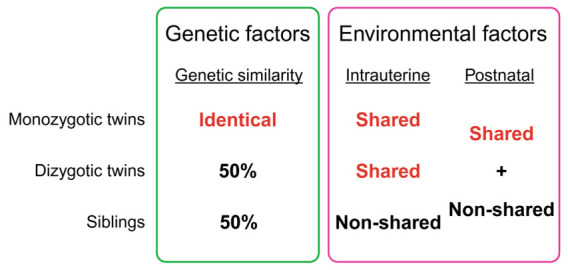
Genetic and environmental factors that contribute to ASD. Conceptual schematic of twin-based estimation of genetic and environmental contributions to ASD liability. Created in BioRender. Kamijo, S. (2026) https://BioRender.com/vpkoxij (accessed on 25 May 2026).

Genetic analyses have been the most successful approach for elucidating the etiology of ASD, yielding robust and reproducible findings. Family based studies, including twin studies, have played a key role in estimating the genetic contribution to ASD risk (Figure 2). Across multiple twin studies, ASD heritability has been estimated to range from 64% to 91%, with shared environmental contributions estimated to be 7–35% [9]. Although these estimates depend on assumptions about population prevalence, genetic factors consistently account for the majority of risk across models.

Recurrence risk refers to the probability that a disorder will occur in another individual, typically a sibling, given that one family member has already been diagnosed with the condition. Factors contributing to ASD can be divided into two components: genetic and environmental. Comparing the recurrence rates between monozygotic and dizygotic twins enables the estimation of genetic contribution by accounting for their shared environmental factors.

The recurrence risk of ASD increases as a function of genetic relatedness, with the highest risk observed in monozygotic twins (recurrence risk relative to control: 153.0), followed by dizygotic twins (recurrence risk relative to control: 8.2) and full siblings (recurrence risk relative to control: 10.3), with progressively lower risks in maternal and paternal half-siblings (recurrence risk relative to control: 3.3 and 2.9, respectively) and cousins (recurrence risk relative to control: 2.0) [10]. The markedly higher recurrence rate among monozygotic twins compared to dizygotic twins suggests a substantial genetic contribution.

Parents from multiplex families (i.e., multiple ASD cases in a family) exhibit more ASD-like personality, language, and socio-behavioral traits than those from simplex families (i.e., single ASD case in a family) and controls [11]. These results suggest that inherited genetic liability is often expressed in subclinical forms. Moreover, recurrence rates are higher in siblings of female probands and in multiplex families [12]. This pattern indicates that these individuals carry a greater inherited genetic burden. Such findings are consistent with a quantitative liability model in which greater genetic load is required to cross the diagnostic threshold.

Evidence from twin and family studies supports a two-layered genetic architecture of ASD, comprising rare variants with large effects and more diffusely inherited liability. High concordance in monozygotic twins is consistent with the contribution of rare, high-impact variants, whereas graded recurrence across genetic distance reflects distributed inherited risk. These observations raise a key question. How do these two layers of genetic risk jointly contribute to ASD susceptibility at the molecular level?

Early genetic studies of ASD relied on linkage analysis and cytogenetic mapping. Subsequent advances in whole-genome and whole-exome sequencing enabled the systematic identification of both copy number variants (CNVs) and rare sequence-level variants that are associated with ASD [13,14]. These sequencing approaches have begun to clarify the genetic architecture of ASD that has been inferred from family-based studies. Autism spectrum disorder-associated variants that have been identified to date are curated in specialized databases, such as Simons Foundation Autism Research Initiative (SFARI) Gene [15].

Although estimates vary depending on datasets and analytical methods, recent large-scale sequencing studies have identified rare variants in 14% of individuals with ASD, approximately half of which are sequence-level variants (52%), nearly half are structural variants, such as CNVs (46%), and a small fraction are mitochondrial variants (2%) [14]. Compared with heritability estimates from family-based studies [9], a substantial portion of genetic risk remains unexplained, highlighting the concept of missing heritability.

### 3.2. Copy Number Variation in ASD

A CNV is a deletion (del) or duplication (dup) of a genomic segment [16]. Genes within the affected region, which can range from several kilobases (kb) to several megabases, typically show alterations of expression because of gene dosage changes. Uniparental disomy, in which both copies of a genomic region are inherited from a single parent, can also disrupt gene expression through abnormal imprinting, although it is not considered a CNV [17]. Because CNVs are more frequent than point mutations and typically involve large genomic segments, they can have a substantial impact on genomic content. The locus-specific mutation rates of CNVs (10^−4^ to 10^−6^ per generation) far exceed those of point mutations (10^−8^ per generation) [18]. Copy number variations add or remove 8–25 kb per transmission, whereas point mutations alter fewer than 100 bp, highlighting the functional impact of CNVs [19].

Although CNVs are found in 10–20% of individuals with ASD, not all of them contribute to ASD risk [20]. Nonetheless, rare CNVs (with <1% allele frequency in the general population), which are either de novo or under strong negative selection, are enriched in ASD [21,22,23]. Certain CNVs such as 15q11–q13 dup, 16p11.2 del/dup, 22q11.2 del, 1q21.1 del/dup, and 7q11.23 del/dup, are recurrently found [24]. Each of these recurrent CNVs occurs in only 0.01–0.33% of ASD cases [14], but they can increase ASD risk approximately 5- to 50-fold, depending on the locus [25,26]. The genes that are affected by these CNVs have been reviewed elsewhere [20,24]. Table 1 summarizes the odds ratio and prevalence in cases (calculated from Abedini et al. [20]) of these major CNV loci. The elevated prevalence figures in the table are partly attributable to the high-sensitivity CNV detection method used in their study.

High-risk CNVs are predominantly de novo and particularly more prevalent in simplex ASD cases [35]. De novo CNVs occur in 10% of simplex cases compared with 3% in multiplex families and 1% in controls [35]. Both deletions and duplications are more frequent in individuals with ASD than in unaffected controls [21,22] and siblings [23]. Because CNVs often span multiple genes, affected individuals typically present with syndromic forms of ASD, accompanied by additional neurodevelopmental or medical features [20]. Notably, many ASD-associated de novo CNVs overlap with regions that are implicated in schizophrenia [25]. This overlap suggests that these CNVs exert nonspecific effects on neurocircuitry [25].

Copy number variations are not uniformly distributed across the genome and are frequently observed at genomic hotspots. Many recurrent CNVs arise from non-allelic homologous recombination, which is mediated by misalignment between low-copy repeats during meiosis [36]. The occurrence of CNVs is associated with maternal age, particularly at low-copy repeat-bound loci, whose high sequence similarity leads to misalignment between chromosomes [37]. In contrast, no clear correlation with paternal age has been observed [38].

The parental origin of de novo CNVs in ASD is not fully understood. In ASD cohorts, approximately 73% of de novo CNVs are estimated to originate from the paternal germline [39]. In contrast, structural variation at the 16p11.2 locus exhibits a maternal bias [40]. These findings suggest that the parental origin of de novo CNVs may vary by locus and remain incompletely understood.

Although rare de novo CNVs with large effect sizes play a dominant role, most CNVs that have been detected in ASD cases are inherited [35]. Both deletions and duplications are enriched in ASD, occurring in both de novo and inherited events [35]. This enrichment of CNVs is particularly pronounced at loci that have been previously implicated in ASD and intellectual disability [22]. Additionally, rare inherited CNVs are more frequent in affected individuals than in unaffected siblings [41]. They substantially overlap with regions that harbor recurrent de novo CNVs, highlighting their functional importance [41].

Inherited CNVs generally confer smaller effect sizes than de novo events but contribute to ASD risk in an additive manner. However, determining their pathological significance is challenging because similar CNVs are also present in the general population at a relatively high frequency [42]. Approximately 65–80% of unaffected individuals carry at least one CNV that is larger than 100 kb [42]. This high background prevalence indicates that most CNVs that have been identified in ASD cases likely have uncertain functional significance, thereby complicating causal interpretations. Consistent with this uncertainty, the SFARI Gene database has discontinued curating CNVs of unclear relevance to ASD risk, and only 17 CNVs were listed in the database as of January 2026.

Animal models with human-equivalent CNVs have been developed to investigate causal effects of gene dosage alterations on neurodevelopment and behavior. Their phenotypic consequences have been comprehensively reviewed elsewhere [24]. Here, we highlight representative examples to illustrate key insights from studies of recurrent, high-confidence CNVs.

Copy number variations in the 15q11-q13 region are recurrently found in ASD cases. This region is subject to genomic imprinting and known as the critical region for Prader–Willi and Angelman syndromes [29]. Mouse models of these CNVs are well-characterized examples of ASD-associated variants. Mice that carry a paternal duplication of this region exhibit ASD-relevant phenotypes, including impairments in social interaction, alterations of ultrasonic vocalizations, and deficits in cognitive flexibility [43]. These behavioral abnormalities are accompanied by low serotonin levels and dysfunction of the dorsal raphe nucleus [44]. A key strength of CNV mouse models is that the duplicated or deleted interval can be narrowed, enabling the functional dissection of individual genes. Using this approach, *Necdin* has been identified as a critical contributor to behavioral and synaptic abnormalities in the paternal 15q11-q13 duplication model. The normalization of *Necdin* copy number rescues both behavioral and synaptic phenotypes [45]. In contrast, maternal duplications of the same region result in distinct phenotypes. *Ube3a*, located in 15q11-q13, is preferentially expressed from the maternal allele in mature neurons [46]. Increasing the *Ube3a* dosage threefold is sufficient to induce ASD-like behaviors in mice [47], illustrating the importance of parent-of-origin effects in this region.

Dosage-sensitive effects have also been extensively characterized at 16p11.2, another recurrent CNV that is strongly associated with ASD. Both deletions and duplications of the mouse region that corresponds to human 16p11.2 result in abnormalities in social behavior, communication, and cognitive function [48]. These changes are accompanied by alterations of brain volume and cortical circuitry [48,49]. Altogether, these findings suggest that recurrent CNVs can affect neural development and behavior in ways that are translatable to humans.

### 3.3. Sequence-Level Variants in ASD

Sequence-level variants in ASD have been extensively studied using whole-genome and whole-exome sequencing [50,51,52,53,54,55]. These variants include single-nucleotide variants (SNVs) and small indels. Variants in protein-coding regions are particularly interesting because their functional consequences are more directly interpretable than those of non-coding variants. For example, mutations that introduce a premature stop codon, frameshift, or abnormal splice site are highly likely to disrupt gene function. Such variants are collectively referred to as likely gene-disrupting mutations. Autism spectrum disorder-associated sequence-level variants are curated in multiple databases, each with distinct inclusion criteria [56].

The human genome acquires approximately 74.5 de novo single-nucleotide mutations per transmission [57]. Rare autosomal coding variants are enriched in ASD and often de novo, as observed for CNVs [58]. Protein-truncating and deleterious missense mutations are preferentially found in genes that are highly intolerant to loss-of-function mutations in individuals with ASD [58].

Sequencing studies of simplex families estimate that approximately 13% of de novo missense and 43% of de novo likely gene-disrupting mutations contribute to sporadic ASD risk [54]. Overall, de novo missense and likely gene-disrupting mutations are estimated to account for approximately 12% and 9% of ASD risk in simplex families, respectively [54]. De novo likely gene-disrupting mutations are more frequently observed in male individuals with lower IQ [54]. Genes that are affected in these individuals overlap significantly with those in females but differ from those that are implicated in higher-IQ males [54]. These observations suggest that a higher genetic burden of deleterious mutations is required for females to exceed the diagnostic threshold. Outside protein-coding regions, analyses of quartet families show no global enrichment of de novo mutations in non-coding regions, which comprise approximately 98.5% of the human genome [59]. However, when non-coding variants are weighted by functional annotations, a modest but statistically significant contribution to ASD risk becomes apparent [59].

Approximately 80% of de novo SNVs originate from the paternal germline, and their number increases proportionally with paternal age in both the general population [51,60] and families with ASD patients [61]. The number of germline mutations doubles every 16.5 years [60]. This pattern is consistent with epidemiological evidence that links advanced paternal age to higher ASD risk [62]. The high replicative activity of male germline cells, which undergo approximately 20–25 divisions per year, is thought to underlie the accumulation of de novo mutations [63].

Despite rapid progress, establishing a definitive list of ASD risk genes remains challenging. Not all variants found in individuals with ASD are necessarily causative. Exome sequencing identified numerous candidate genes, but gene lists vary considerably depending on statistical thresholds and inclusion criteria. Some databases incorporate genes that are associated with syndromic ASD or broader neurodevelopmental disorders. Given that up to 40% of individuals with ASD also have intellectual disability, a subset of variants may act through shared neurodevelopmental pathways rather than ASD-specific mechanisms [7]. In addition, individual sequence-level variants are often too rare to be amenable to robust statistical analysis. Nonetheless, some syndromic forms of ASD are clinically validated and provide key insights into its pathogenesis. To recapitulate human clinical findings, a wide range of knockout and knock-in mouse models have been engineered. Table 2 summarizes frequent syndromic ASD caused by single gene disruption.

Inherited sequencing-level variants with small to moderate effect sizes also contribute to ASD risk. Studies of simplex and multiplex families have shown that rare inherited variants, including likely gene-disrupting mutations, are enriched in individuals with ASD [69,70]. Although inherited variants generally confer smaller effect sizes than de novo variants, they exert additive effects that help establish a predisposing genetic background [69]. Relative risk decomposition across four genetic classes—de novo and inherited CNVs and SNVs—revealed a gradient of effect size, with de novo CNVs conferring the highest odds ratio (OR = 2.05), followed by de novo disruptive SNVs (OR = 1.72), inherited CNVs (OR = 1.23), and inherited disruptive SNVs (OR = 1.11) [69]. Genome-wide association studies have successfully identified common risk alleles for many diseases [71]. In ASD, however, only five genome-wide significant loci have been identified, all located in non-coding regions [72].

These genetic findings support a model in which ASD risk arises from rare, large-effect variants (“rare big hits”), superimposed on a diffuse burden of inherited variants that collectively confer susceptibility.

### 3.4. Other Forms of Genetic Contribution and Convergent Biological Pathways

Copy number variants and sequence-level variants, whether de novo or inherited, account for only a fraction of the total heritable risk of ASD [14]. The gap between variant-based risk estimates and heritability that has been inferred from twin studies suggests substantial missing heritability. Compared with coding variants, the functional consequences of non-coding mutations are more difficult to predict, and their typically small effect sizes have hindered discovery. Nevertheless, regulatory variants that influence gene expression levels or spatiotemporal patterns contribute to ASD risk [73]. Another group reported a genome-wide significant polymorphism in a gene-poor region of chromosome 5p14.1 [74]. From this locus, a non-coding RNA named *MSNP1AS* is transcribed, which binds to *MSN* (moesin) mRNA. Elevated expression of *MSNP1AS* has been observed in postmortem ASD brains, implying its involvement in ASD pathogenesis by modulating moesin protein expression [74].

As the number of ASD-associated genes continues to grow, significant effort has been directed toward identifying convergent biological pathways. Genes that are disrupted by de novo CNVs and loss-of-function SNVs cluster within networks that are involved in neuronal signaling and development, synaptic function, and chromatin regulation [75]. ASD risk genes form gene modules that are associated with early transcriptional regulation and synaptic development [76].

Beyond defining convergent biological pathways, recent studies clarified the cell types and developmental stages in which ASD risk genes are most prominently expressed and functionally engaged. High-confidence ASD risk genes show preferential expression in midfetal layer 5/6 projection neurons [26], glutamatergic projection neurons and superficial-layer cortical neurons [76], and both excitatory and inhibitory neurons [58]. Single-nucleus transcriptomic studies of postmortem brains revealed the preferential dysregulation of synaptic signaling pathways in upper-layer excitatory neurons, accompanied by alterations of microglial molecular states [77]. Although overall shifts in cell-type composition appear to be modest, activated microglia, astrocytes, and somatostatin interneurons are preferentially localized to superficial cortical layers [78].

Epigenetic dysregulation represents an additional layer of risk. Epigenetic modifications serve as a critical interface between environmental factors and genetic predispositions. DNA methylation analyses of monozygotic twins identified ASD-associated differentially methylated regions that correlated with autistic trait severity [79]. Postmortem brain studies reported alterations of the expression of activity-dependent neuronal genes and immune-response genes [80,81]. Genome-wide methylation analyses further demonstrated that ASD-associated co-methylation modules are enriched for synaptic, neuronal, γ-aminobutyric acid (GABA)ergic, and immune-related gene networks [82]. Genes subjected to differential methylation (i.e., hypermethylation or hypomethylation) in individuals with ASD are reviewed here [83].

Genetic studies have identified epigenetic regulators as causative genes for syndromic forms of ASD. A classic example is Rett syndrome. Although Rett syndrome is a distinct clinical diagnosis and is excluded from ASD in the DSM-5 classification, it provides important insights into the contribution of epigenetic mechanisms to ASD development. In most cases of Rett syndrome, mutations in the *MECP2* gene are found. *MECP2* encodes a protein that binds to methylated DNA to repress transcription [84]. Notably, *MECP2* duplication also leads to ASD-like behavioral deficits, a condition known as *MECP2* duplication syndrome [85]. This dosage-sensitive phenomenon indicates that epigenetic transcriptional control requires a specific homeostatic window. Any deviation from this optimal range, whether through an increase or a decrease in gene expression, results in unfavorable neurodevelopmental outcomes. The MECP2 protein has also been reported to have additional functions, including the regulation of microRNAs [86] and the preferential repression of long genes [87]. Similarly, disruptions in other chromatin-related factors, such as the chromatin remodeler *CHD8* or the histone H3K9 methyltransferase *EHMT1*, are reported to elicit social behavioral abnormalities characteristic of ASD [88,89].

### 3.5. Functional Insights from Knockout and Knock-In Mouse Models

Since the initial characterization of neuroligin-knockout mice [90,91], numerous ASD risk genes that have been identified in humans have been functionally investigated using knockout approaches [92]. These models encompass both syndromic and non-syndromic forms of ASD. As of January 2026, phenotypic data from mouse models that correspond to 269 ASD-associated genes are cataloged in the SFARI Gene database [15]. Although individual knockout models exhibit gene-specific phenotypes, they establish whether the disruption of a given gene that has been identified in humans is sufficient to produce ASD-relevant behavioral and neurobiological features. Furthermore, with the advancement of genome engineering techniques, an increasing number of knock-in mouse models carrying clinical mutations identified in individuals with ASD have been generated (see also Table 2). The phenotypic data of such knock-in mouse models are also curated in the database [15], providing critical insights into mutation-specific phenotypes.

In summary, twin- and family-based analyses consistently demonstrate a substantial genetic contribution to ASD risk. This genetic architecture consists of rare high-impact variants and a background burden of predominantly inherited variants with smaller effects that collectively influence overall susceptibility.

## 4. Environmental Factors

Although genetics accounts for a substantial portion of ASD risk, environmental factors, particularly those that act during prenatal and perinatal periods, also contribute, generally with modest effect sizes [93,94,95]. Epidemiological studies have identified risk factors, such as maternal immune activation, preterm birth, perinatal hypoxia, in utero drug exposure, and maternal metabolic conditions. Directly comparing the effect sizes of various environmental factors is challenging due to the significant heterogeneity in study populations and exposure assessment methods. The relative risks associated with various environmental factors have been comprehensively reviewed [96].

Importantly, environmental exposures do not lead to ASD in all individuals. Instead, these factors interact with underlying genetic susceptibility to reshape the epigenetic landscape, ultimately influencing neurodevelopmental outcomes [97]. Despite the theoretical appeal of the gene × environment interaction model, empirical validation in ASD remains challenging due to the following reasons: (1) inherent polygenicity of the disorder, (2) temporal sensitivity of environmental insults, (3) requirement for larger sample sizes to isolate a small interaction term from main effects (i.e., genetic or environmental effects), and (4) clinical heterogeneity of the disorder. Unfortunately, gene × environment interaction is sometimes used as a catch-all term for the variance that cannot be explained by genetic or environmental factors independently. Given that it is a vast topic in its own right, please refer to the following reviews for a comprehensive discussion [97,98]. This topic will be discussed again in Section 9.

### 4.1. Maternal Immune Activation and Immune System Alterations in ASD

Maternal immune activation represents one of the most extensively studied environmental risk factors for ASD [99,100]. The idea that maternal infection can influence neurodevelopment was first proposed in the context of schizophrenia following the 1957 influenza epidemic [101] and later extended to ASD. Large epidemiological studies have reported associations between maternal viral or bacterial infections during pregnancy and an increase in ASD susceptibility in offspring [102,103]. A meta-analysis estimated a 13% increase in risk following maternal infection, with greater effects among hospitalized mothers [104]. Similarly, maternal infection or fever has been linked to a 32% increase in ASD odds, without clear evidence of trimester-specific vulnerability in another meta-analysis [105]. In a case–control study, high maternal cytokine levels during midgestation are associated with roughly a 50% increase in ASD susceptibility [106]. These findings suggest that maternal immune activation itself, rather than pathogen-specific effects, can alter neurodevelopment.

Maternal autoimmune conditions are also associated with a modest increase in ASD risk. Children of mothers with autoimmune disease had a 21% higher risk of neurodevelopmental disorders in a large epidemiological cohort [107], and a meta-analysis estimated a 34% increase specifically for ASD [108]. Additionally, in a case–control study, mothers of children with ASD are four times more likely to harbor anti-brain autoantibodies than mothers of typically developing children [109].

However, the observed increase in ASD risk that is associated with maternal immune activation or autoimmune conditions remains modest. The majority of exposed individuals do not develop ASD, emphasizing the importance of gene × environment interactions. These apparent associations may stem from a genetic predisposition toward immune hyper-responsiveness, rather than from the inflammatory insults themselves.

Beyond maternal influences, individuals with ASD exhibit heightened immune activity. Case–control studies and a subsequent meta-analysis have demonstrated that plasma levels of proinflammatory cytokines and chemokines are elevated and correlate with the severity of behavioral impairment [110,111,112]. Postmortem case–control studies revealed neuroinflammation in the cerebral cortex, white matter, and cerebellum, with microglial and astroglial activation [113]. These findings suggest that sustained immune activation is a recurrent feature of ASD.

Moreover, as demonstrated in the following case–control studies, anti-brain autoantibodies are more frequently detected in ASD. Shared immunoreactivity between mothers and children with ASD has been observed against prenatal rat brain proteins but not against postnatal or adult brain antigens [114]. In children with ASD, plasma shows higher immunoreactivity to cerebellar proteins compared with controls (21% vs. 2%), including strong reactivity to macaque cerebellar Golgi cells, and the presence of anti-cerebellar antibodies has been independently confirmed [115,116].

Studies have investigated how maternal immune activation influences fetal brain development, leading to long-lasting behavioral and neurobiological outcomes [100,117]. Although maternal immune cells do not cross the placental barrier, cytokines and antibodies can reach the fetus and act as mediators, providing a plausible biological link between maternal inflammation and alterations of neurodevelopment.

Animal models have been extensively used to study the causal link between maternal immune activation and ASD-relevant behaviors of the offspring, because human epidemiological studies are limited to identifying associations. Commonly used rodent models involve the maternal administration of polyinosinic-polycytidylic acid (poly[I:C]) and lipopolysaccharide, which mimic viral and bacterial infections, respectively [118,119,120]. These paradigms reliably induce transient maternal cytokine responses and produce behavioral phenotypes in offspring that are relevant to ASD. Maternal poly(I:C) exposure is sufficient to induce ASD-relevant behavioral phenotypes in mice, including low sociability and impairments in prepulse inhibition. These effects are mediated by maternal cytokine signaling. For example, the coadministration of an interleukin-6 (IL-6)-neutralizing antibody abolished poly(I:C)-induced behavioral abnormalities [121].

The relevance of maternal immune activation is not restricted to rodents. Maternal poly(I:C) administration also induces social deficits in marmosets [122]. The mechanisms that link maternal immune activation to alterations of social behavior are conserved across species and support their translational validity.

Subsequent rodent studies demonstrated that poly(I:C)-induced behavioral and cortical abnormalities depend on transplacental IL-17A that is produced by maternal Th17 cells. These phenotypes can be rescued by IL-17A neutralization or by deleting maternal Th17 cells, indicating a pivotal role for IL-17A [123].

Prenatal immune activation can induce long-lasting changes in the offspring’s immune function. Rodent studies demonstrate that prenatal poly(I:C) exposure leads to persistent alterations of cytokine expression in the brain and periphery [124], cellular immunity [125]. Transcriptomic profiles and phagocytic function of microglia, which are essential for normal neurodevelopment, are altered in poly(I:C)-exposed mouse offspring [126].

Animal models involving in utero exposure to maternal autoantibodies have provided critical insights into the immune-mediated etiologies of ASD. Prenatal exposure to immunoglobulin obtained from mothers of children with ASD has been shown to induce altered sociability and elevated anxiety in mice [127]. Similar prenatal immunoglobulin treatment led to stereotypies and hyperactivity [128], as well as altered brain growth and social behavior in rhesus monkeys [129]. Among anti-brain positive mothers of children with ASD, approximately 40% are positive for anti-CASPR2 antibody [130]. In utero exposure to monoclonal anti-CASPR2 antibody cloned from such mothers was sufficient to induce impairments in sociability, flexible learning, and repetitive behavior in mice, in a male-specific way [130]. These results collectively indicate that maternal autoantibodies can drive ASD pathogenesis.

### 4.2. Preterm Birth and Perinatal Hypoxia

Preterm birth is consistently associated with a higher risk for ASD. In a meta-analysis, approximately 7% of preterm-born children are later diagnosed with ASD, with progressively higher risk at earlier gestational ages [131]. In a Swedish cohort study, adjusted prevalence ratios increase in a graded manner, reaching 3.87 for extremely preterm birth (22–27 weeks), 1.65 for very to moderate preterm birth (28–33 weeks), 1.25 for late preterm birth (34–36 weeks), and 1.12 for early term birth (37–38 weeks), compared with full-term birth [132]. Similar gestational age-dependent gradients have been reported in Nordic countries and Taiwan population-based cohorts [133,134].

Neuroanatomical studies in individuals who are born preterm demonstrate widespread white matter dysmaturation, alterations of cortical thickness, and disruptions of thalamocortical connectivity [135,136]. The delayed maturation of inhibitory circuits has also been described, suggesting that preterm birth can interfere with multiple aspects of brain development and lead to long-lasting neuroanatomical changes [137]. However, co-occurring intrauterine infection, a major cause of premature delivery, makes disentangling the independent contribution of preterm birth to ASD risk difficult.

Similar to maternal immune activation, epidemiological studies on preterm birth only demonstrate an association with ASD. Experimental animal models that isolate causal effects of preterm birth per se remain limited. Most preterm animal paradigms rely on inflammatory or hormonal manipulations that may directly influence fetal brain development, complicating mechanistic interpretation. One proposed pathophysiological mechanism that links preterm birth to ASD risk is the premature loss of maternal- and placental-derived factors that are normally involved in late gestational brain development, together with early exposure to extrauterine conditions [138].

Meta-analyses have also implicated perinatal hypoxia as a risk factor for ASD. Low 5 min Apgar scores, a proxy for compromised neonatal oxygenation, are linked to a higher risk for ASD and intellectual disability, even among term-born infants [139,140]. Another meta-analysis reported that prolonged labor is associated with a 1.77-fold increase in ASD risk, although the results were inconsistent across studies [141]. These observations suggest that hypoxic insults may contribute to ASD risk independent of preterm delivery. Nevertheless, like preterm birth, perinatal hypoxia is not specific to ASD. It confers vulnerability across various neurodevelopmental and psychiatric conditions, such as attention-deficit hyperactivity disorder and schizophrenia [142,143].

### 4.3. In Utero Drug Exposure

Prenatal drug exposure constitutes a distinct class of environmental risk factors. Although such exposures account for only a small fraction of ASD cases, certain agents, particularly valproic acid (VPA) and selective serotonin reuptake inhibitors (SSRIs), have been examined extensively.

Valproic acid, a widely used antiepileptic drug, shows a consistent association with high ASD risk [144]. Gestational exposure to VPA was linked to a 2.9-fold higher hazard of ASD in a Danish population-based cohort with no clear difference between early and late pregnancy exposure [145]. A meta-analysis similarly reported a 2.8-fold higher risk following prenatal VPA exposure [146]. They also showed weaker and less consistent associations for other antiepileptic drugs, such as carbamazepine and oxcarbazepine [146].

While human studies show associations, the causal impact of in utero VPA exposure has been directly demonstrated using experimental animal models. In utero exposure to VPA induces ASD-relevant behavioral phenotypes, including impairments in social interaction in rodents and nonhuman primates [147,148,149,150]. These observations suggest that the developmental pathways disrupted by prenatal VPA exposure are conserved across species.

Mechanistically, VPA is thought to act through epigenetic mechanisms by inhibiting histone deacetylases (HDACs). Consistent with this, valpromide, a structural analog that lacks HDAC inhibitory activity, does not reproduce ASD-like behavioral phenotypes [151]. In contrast, the HDAC inhibitor trichostatin A partially elicits similar behavioral alterations in mice [152]. The molecular changes that are induced by prenatal VPA exposure are diverse and have been reviewed elsewhere [153].

Animal studies using naïve subjects provided evidence for a direct causal contribution of prenatal VPA exposure by isolating the drug’s effects from any underlying pathology. In clinical settings, however, the presence of maternal epilepsy remains a potential confounding factor that complicates the interpretation of ASD risk.

In contrast to the robust and consistent associations observed with prenatal VPA exposure, the association between prenatal SSRI exposure and ASD remains controversial. Although an early population-based case–control study reported an approximately 2.2-fold higher risk, the finding was confounded by maternal psychiatric illness, which itself carries heritable ASD liability [154]. Indeed, in a Danish cohort study, SSRI use during pregnancy was not associated with a higher ASD risk, whereas SSRI use prior to pregnancy was linked to a 1.46-fold increase [155]. Meta-analytic evidence indicates that the modest association largely disappears when analyses are restricted to mothers with psychiatric disorders [156]. These results suggest that maternal psychiatric illness may account for a substantial proportion of the observed association, rather than SSRI exposure itself.

While epidemiological evidence remains inconclusive, experimental animal studies have actively tested the consequences of disrupting serotonergic pathways during development. Gestational and early postnatal exposure to fluoxetine, an SSRI, reduces sociability, impairs social novelty recognition, and disrupts working memory in rodents [157,158]. These behavioral alterations are accompanied by persistent increases in the intrinsic and serotonin-induced excitability of fast-spiking interneurons in the medial prefrontal cortex [158]. These findings indicate that prolonged serotonergic modulation during sensitive developmental periods can influence neurocircuit maturation.

Given their widespread use and the potential for population-level impact, prenatal exposure to more commonly used medications is also of interest. Paracetamol, one of the most commonly used medications during pregnancy, has not been associated with an increased risk of ASD in a meta-analysis [159].

### 4.4. Maternal Metabolic Conditions, Stress, and Pollutant Exposure During Pregnancy

Maternal diabetes has been associated with a modest increase in ASD risk. Meta-analyses reported a pooled relative risk of 1.48 in cohort studies, including both pregestational and gestational diabetes, with pooled ORs that ranged from 1.42 for gestational diabetes alone to 1.72 for any maternal diabetes in case–control studies [160,161]. Proposed mechanisms include maternal hyperglycemia-induced oxidative stress, inflammation, and alterations of fetal brain insulin signaling that may disrupt neurodevelopment during sensitive periods [162].

Prenatal stress is known to interfere with normal neurodevelopment, exerting long-lasting effects on the offspring [163]. Extensive epidemiological evidence has linked prenatal exposure to stressful life events with an increased risk of various neuropsychiatric conditions, including ASD [163]. An early study identified a higher incidence of prenatal stressors at 21–32 weeks gestation, with a peak at 25–28 weeks in cases of ASD [164]. A meta-analysis demonstrated that prenatal stress exposure is associated with an increased risk of ASD (pooled OR 1.64) [165].

Prenatal exposure to severe objective stress from hurricanes was significantly associated with an increased risk of ASD in the offspring, particularly when the exposure occurred near middle or end of gestation [166]. Exposure to maternal bereavement yielded mixed results. In a Danish cohort, no association was observed between maternal bereavement and ASD [167]. In contrast, a subsequent Swedish cohort study reported that third-trimester exposure increased the risk of ASD (adjusted hazard ratio 1.58) [168].

Dysregulation of hypothalamus–pituitary–adrenal axis is proposed as an underlying biological mechanism linking prenatal stress to altered neurodevelopment [169,170]. Maternal stress can downregulate placental 11β-hydroxysteroid dehydrogenase type 2 (11β-HSD2), an enzyme that normally metabolizes cortisol into its less active form, cortisone, thereby shielding the fetus from excess maternal cortisol [169]. When this placental barrier is compromised, excess cortisol gains access to the fetal circulation and binds to fetal glucocorticoid receptors. This exposure triggers persistent epigenetic modifications, such as altered DNA methylation of stress-related genes, which may permanently change the offspring’s neurodevelopmental trajectory [169].

Prenatal exposure to air pollutants is associated with an elevated ASD risk in multiple studies [171]. Two US studies suggested that exposure during the third trimester is the most relevant [172,173]. However, European twin study and population-based cohort study showed no association between air pollutant with ASD [174,175]. A recent meta-analysis demonstrated that prenatal exposure to air pollutants is associated with slight but significantly increased odds ratio (1.06 for particulate matter 2.5, and 1.02 for nitrogen dioxide) across studies [176].

However, the reported prevalence of ASD is highly influenced by detection thresholds and diagnostic accessibility as we discussed in epidemiology section. Although such confounding factors are carefully taken into account, residual unadjusted factors may account for the observed association. In addition, the supporting biological mechanisms remain limited. Future epidemiological studies from diverse geographical regions, particularly from non-US countries, are warranted to validate these findings. Furthermore, efforts to identify plausible biological mechanisms linking prenatal air pollutant exposure to neurodevelopmental alterations are essential.

In summary, epidemiological studies have identified a wide range of environmental factors that are associated with small but significant increases in ASD risk. Since these factors are derived from epidemiological observations, common genetic liabilities shared between mother and offspring remain a major confounding factor. Although transient exposure during sensitive developmental periods may influence neurodevelopmental trajectories, the observed risk increases are generally modest. Indeed, the majority of exposed individuals do not develop ASD. Collectively, these findings emphasize the multifactorial nature of ASD etiology and the significance of gene × environment interactions in determining individual susceptibility.

## 5. Excitation/Inhibition Imbalance and the GABAergic System

### 5.1. Molecular Evidence of Shifted Excitation/Inhibition Balance in Individuals with ASD

An imbalance between excitation and inhibition (E/I) has been proposed as a potential pathophysiological mechanism of ASD [177,178]. Several lines of evidence have been interpreted in support of this hypothesis. In a meta-analysis, individuals with ASD exhibit a higher prevalence of epilepsy (approximately 10%), a finding that suggests an increase in network excitability [179]. At the structural level, postmortem studies have reported an increase in the density of excitatory dendritic spines across multiple cortical regions, findings that are compatible with alterations of excitatory connectivity [180].

Converging molecular and neurochemical evidence further implicates impairments in inhibitory signaling. Postmortem studies have revealed that expression of the GABA-synthesizing enzymes glutamic acid decarboxylase 65 (GAD65) and GAD67 is lower in the cerebellum [181], and GABA receptor subunits are downregulated in both the cerebral cortex and cerebellum [181,182]. In vivo imaging studies have also reported reduced GABA_A_ receptor binding in the superior and medial prefrontal cortex of children with ASD [183]. These results suggest that inhibitory tone is diminished in the ASD brain. Altogether, these observations underscore a relative shift toward an increase in excitatory influence, although regional and developmental heterogeneity has been reported.

Genomic studies provide complementary support for the E/I imbalance hypothesis, as ASD risk genes are enriched in pathways that are related to synaptic function and GABAergic neurotransmission [26,58]. These findings implicate altered neurotransmission as a convergent biological feature of ASD, although it may represent a secondary consequence rather than the primary etiology.

### 5.2. Functional Evidence of Shifted Excitation/Inhibition Balance in Individuals with ASD

Human functional studies using a case–control design further provide the supporting evidence. Individuals with ASD exhibit alterations of gamma-band activity on electroencephalography. Visually induced gamma responses are attenuated and delayed in adults with ASD [184]. Such gamma-band abnormalities are commonly attributed to the dysfunction of parvalbumin-positive interneurons [185]. Early sensory processing is likewise affected in ASD. The P100 component of the visual evoked potential shows lower amplitude and higher trial-to-trial variability [186]. The 40 Hz auditory steady-state response is lower and less phase-consistent [187]. Given the central role of GABAergic transmission in generating gamma oscillations and shaping early sensory responses, these findings suggest that the molecular alterations in inhibitory signaling observed in individuals with ASD have tangible functional consequences at the circuit level.

Neurometabolite measurement with magnetic resonance spectroscopy is of particular interest, as it provides non-invasive insight into altered E/I balance in the ASD brain [188]. However, findings have been inconsistent across different metabolites and brain regions. A recent meta-analysis demonstrated that GABA is significantly lower in individuals with ASD across studies [189]. In contrast, glutamate levels were comparable to controls. These meta-analytic findings offer compelling evidence that the GABAergic system is primarily impaired in ASD. Inspired by these neurometabolite data, clinical trials aiming to modulate the GABAergic system have been conducted. However, enhancing GABAergic signaling via arbaclofen, a GABA_B_ receptor agonist, showed limited clinical efficacy in both fragile X syndrome [190] and autism spectrum disorder [191].

### 5.3. Causal Insights from E/I Manipulation in Animal Models

The E/I imbalance hypothesis has been extensively examined in animal models, primarily because these systems allow for the direct testing of whether such imbalances play a causal role in ASD-like phenotypes. Acutely increasing excitatory tone in the medial prefrontal cortex is sufficient to induce behavioral impairments, including lower sociability and higher high-frequency rhythmicity in local cortical circuits [192]. Conversely, restoring E/I balance either by enhancing the excitability of parvalbumin-positive interneurons or by reducing the excitability of pyramidal neurons ameliorates ASD-relevant phenotypes in contactin-associated protein-like 2 (*CNTNAP2*)-deficient mice [193]. Even transient shifts in cortical excitability can concurrently alter behavioral output, underscoring the real-time sensitivity of prefrontal circuits to E/I perturbation.

The direct disruption of GABAergic neurotransmission similarly produces ASD-like phenotypes. Parvalbumin-deficient mice exhibit impairments in sociability and cognitive inflexibility [194], and GABA_A_ receptor subunit β3 (*GABRB3*) knockout mice exhibit social deficits accompanied by hypoplasia of the cerebellar vermis [195]. Forkhead box protein G1 (*FOXG1*) heterozygous mice serve as a model for *FOXG1* syndrome, a severe neurodevelopmental disorder frequently accompanied by autistic features in humans. In this model, increasing inhibitory tone by the transplantation of GABAergic interneurons into the medial prefrontal cortex ameliorates social impairments [196]. In contrast, decreasing inhibitory tone by the mutation of the *GAD2* gene (which encodes GAD65) exacerbates these behavioral deficits [196]. Notably, these effects are observed only when the E/I imbalance is corrected before the second postnatal week, suggesting a critical developmental time window during which E/I interactions shape circuit maturation [196]. Thus, the developmental disruption of E/I balance can exert long-lasting effects on neurocircuits and behavior.

### 5.4. E/I Balance in ASD Mouse Models and Its Developmental Regulation

While acute and developmental manipulations demonstrate the causal sufficiency of E/I imbalance, similar neurophysiological signatures are observed in models that capture the diverse etiologies of ASD. Alterations of inhibitory interneuron populations have been reported in multiple ASD models, including VPA-exposed mice and neuroligin-3-deficient mice [197]. These findings indicate that inhibitory circuit dysfunction may recur across etiologically distinct conditions.

Shifted E/I balance toward excitation is reported in multiple syndromic forms of ASD [178,198,199]. In individuals with fragile X syndrome, circuit hyperexcitability is observed as an elevated prevalence of epilepsy (12%) [200]. This effect is thought to be mediated by aberrant inhibitory circuits [201]. High prevalence of epilepsy (80%) is also reported in individuals with Angelman syndrome [202]. Angelman syndrome is caused by either loss of *Ube3a* expression or chromosomal deletion of 15q11-13 region (70% of cases) [203]. In a mouse model, *Ube3a* deficiency decreases tonic GABAergic inhibition through reduced degradation of GABA transporter 1 (GAT-1) protein [204]. The deletion type typically exhibits more severe epileptic phenotypes, and this is attributed to the co-deletion of GABA_A_ receptor genes (*GABRB3*, *GABRA5*, and *GABRG3*) in the affected region [203].

These recurrent findings suggest that diverse insults might converge on a common developmental program. In particular, the developmental trajectory of E/I balance is shaped by the maturation of GABAergic signaling. During early life, GABA is depolarizing, owing to high intracellular chloride concentrations in rodents and humans [205]. Postnatal changes in the expression of chloride transporters, such as Na-K-2Cl cotransporter-1 (*NKCC1*) and K-Cl cotransporter 2 (*KCC2*), drive the transition to hyperpolarizing inhibition. In a mouse model of Dravet syndrome, a developmental epileptic encephalopathy that is frequently accompanied by social and cognitive impairments, pharmacological acceleration of this GABA polarity shift improves social interaction deficits and reduces hyperactivity [206]. These findings indicate that the timing of inhibitory maturation can modulate behavioral outcomes.

Collectively, evidence from molecular, genetic, and circuit-level functional studies supports the presence of E/I imbalance in individuals with ASD and in various animal models. Both acute manipulations and developmental alterations of E/I balance, especially those that favor greater excitation, are sufficient to induce social and cognitive abnormalities in rodents. However, remaining unclear is whether E/I imbalance is a primary pathogenic mechanism or downstream convergence point in humans. Identifying the most vulnerable neurocircuits and elucidating how region- and stage-specific shifts in E/I balance result in persistent behavioral abnormalities are essential for refining this model.

## 6. Brain–Gut Hypothesis

### 6.1. Insights from Animal Models

The brain and gut communicate through multiple pathways, including endocrine signaling, autonomic nervous system pathways, immune mechanisms, and microbiota-derived metabolites [207]. Animal studies demonstrate that gut microbiota can influence social behavior and other ASD-relevant phenotypes. A notable example is germ-free mice exhibit impairments in social behavior and an increase in repetitive behaviors, which are partially ameliorated by post-weaning bacterial colonization [208].

Brain–gut interaction is dysregulated in etiologically distinct ASD models. In the maternal immune activation model, offspring exhibit alterations of the gastrointestinal barrier alongside ASD-like behaviors [209]. Both histological and behavioral phenotypes can be mitigated by treatment with specific bacterial strains, such as *Bacteroides fragilis* [209]. Furthermore, the colonization of germ-free mice with microbiota that were derived from individuals with ASD reproduced ASD-like behaviors across donors [210]. In contrast, the transfer of microbiota from neurotypical controls failed to induce such phenotypes, suggesting that the observed behavioral changes are specific to the ASD-associated flora [210]. Although these findings are derived from animal models, they suggest that the gut microbiota may not only contribute to the pathogenesis of ASD but also serve as a potential therapeutic target.

### 6.2. Clinical Evidence in Human Subjects

Inspired by insights from animal models, clinical research in humans has gained significant momentum in recent years. In humans, individuals with ASD frequently report gastrointestinal problems, which have been associated with greater behavioral difficulties, including irritability, social withdrawal, stereotypy, and hyperactivity [211]. Gastrointestinal problems in ASD are often accompanied by alterations of fecal microbial composition [212]. However, findings vary across studies regarding which bacterial groups differ and how overall microbiota composition is altered [213].

Building on observations from germ-free animal models, fecal microbiota transfer studies have been conducted in individuals with ASD. Open-label studies have reported improvements in gastrointestinal symptoms and ASD-relevant behavioral measures [214], but these effects appear to be transient, and evidence from randomized controlled trials remains limited [215,216].

Several considerations temper the interpretation of these human findings as evidence for a primary etiological role in ASD and significant concerns have been raised about this brain–gut interaction hypothesis [217]. Establishing causality in humans remains challenging because longitudinal data prior to symptom onset are scarce. Consequently, it remains unclear whether microbiota alterations act as primary drivers or merely reflect secondary adaptations to distinct dietary patterns and medication exposure. Furthermore, the significant heterogeneity across studies regarding microbial composition, intestinal pathology, and intervention efficacy precludes a unified consensus. Finally, while germ-free and microbiota-transfer paradigms offer powerful mechanistic insights, they represent highly simplified experimental systems. Their translational relevance to the complexity of human development remains to be fully elucidated.

Overall, current evidence indicates that gut microbiota can influence neurodevelopment and behavior under specific conditions. However, it remains to be determined whether these findings translate into a central pathogenic mechanism for ASD in humans.

## 7. Neuromodulators

### 7.1. Serotonin

Serotonin (5-hydroxytryptamine [5-HT]) has long been implicated in ASD [218,219,220]. In case–control studies, high peripheral serotonin levels were first described as a potential biomarker of ASD in a subset of individuals with ASD in the 1960s [221,222]. Subsequent meta-analyses reported hyperserotonemia in approximately half of published studies, although associations with behavioral severity remain variable [223].

Serotonin biology in ASD is complex because peripheral and central serotonin pools are largely distinct. Approximately 95% of serotonin is synthesized in enterochromaffin cells in the intestine [224], whereas central serotonin is produced in raphe nuclei of the midbrain, which project widely throughout the brain. During pregnancy, the placenta also contributes serotonin to the developing forebrain [225].

Beyond peripheral findings, case–control neuroimaging studies point to alterations of central serotonergic function in individuals with ASD. Positron emission tomography studies show that cortical serotonin synthesis capacity follows an atypical developmental trajectory in children with ASD [226]. Reduced serotonin transporter binding has also been observed across multiple brain regions in adults with ASD, and its availability correlates with social cognitive performance [227]. In parallel, gain-of-function mutations of the serotonin transporter were identified in a subset of individuals with ASD [228].

Experimental animal studies indicate that serotonin plays a critical role in neural circuit maturation and the development of ASD-relevant behavioral phenotypes. In mice, postnatal reductions in serotonin levels trigger barrel formation in the somatosensory cortex [229]. This maturation process is accelerated following preterm birth but can be normalized by increasing serotonin levels, suggesting that serotonin plays an important role for perinatal circuit refinement [229].

Direct manipulation of serotonin signaling is reported to induce ASD-like behavioral phenotypes. Mice that carried the Ala56 gain-of-function mutation exhibited ASD-like behaviors, peripheral hyperserotonemia, and alterations in the firing of raphe 5-HT neurons [228]. Aberrant serotonin signaling is also observed in an etiologically distinct mouse model. In the paternal 15q11-13 duplication model, mice exhibit low brain serotonin levels and functional abnormalities in the raphe nuclei on positron emission tomography imaging, accompanied by ASD-like behaviors [44]. Consistent with alterations of serotonergic modulation, sensory responses in the barrel cortex are spatially more diffuse than in controls [44].

### 7.2. Oxytocin and Vasopressin

Oxytocin is an evolutionarily conserved nine-amino-acid peptide hormone that is traditionally recognized for its roles in uterine contraction and lactation. Its involvement in social behavior emerged from seminal studies of monogamous voles that demonstrated a role in pair bonding and social attachment [230,231], followed by human experiments that found trust-promoting effects [232].

Alterations of oxytocin signaling have been investigated in ASD. Early case–control studies reported low peripheral oxytocin levels in children with ASD [233], although subsequent findings have varied across studies. Meta-analyses indicate that peripheral oxytocin concentrations are lower in children with ASD but not in adolescents or adults [234,235]. Genetic evidence provides additional support. A meta-analysis identified a polymorphism of the oxytocin receptor (*OXTR*) gene that was associated with a modest increase in ASD risk (OR = 1.31) [236].

The therapeutic potential of intranasal oxytocin has been explored. Intranasal oxytocin administration improved the retention of affective speech comprehension [237]. It also enhanced emotion recognition on the Reading the Mind in the Eyes Task [238]. Larger randomized controlled trials, however, produced variable outcomes. A 4-week trial in children with autism reported modest improvements in social functioning, particularly among individuals with low baseline oxytocin levels [239]. In contrast, another double-blind study found no significant change in social reciprocity after 6 weeks of treatment, despite minor effects on gaze fixation and repetitive behaviors [240]. A possible non-linear dose–response relationship has also been proposed [241]. Overall, clinical evidence indicates that oxytocin exerts modest and context-dependent effects on social symptoms.

Animal models provide more consistent evidence of a role for oxytocin signaling in social behavior. Oxytocin knockout mice exhibit impairments in social memory [242], and oxytocin receptor knockout mice exhibit deficits in social discrimination and greater aggression [243]. Region-specific receptor deletions further reveal circuit-level specificity. For instance, forebrain deletion leads to impaired social discrimination [244], while hippocampal CA2/CA3a-specific deletion results in the disruption of long-term social recognition memory [245]. Oxytocin receptor-expressing neurons are broadly distributed throughout the brain, with enriched expression in nuclei that are implicated in social processing [246].

The contribution of vasopressin to ASD has also gained significant attention. Vasopressin is a nine-amino acid hormone structurally similar to oxytocin, with which it shares seven residues. In a case–control study, children with ASD exhibit lower levels of arginine vasopressin in the cerebrospinal fluid [247]. Furthermore, polymorphisms in an arginine vasopressin receptor 1a (*AVPR1a*) gene are associated with ASD susceptibility [248].

The clinical efficacy of vasopressin has also been evaluated in a randomized controlled trial. Four weeks of intranasal vasopressin administration significantly improved social communication in children with ASD [249]. These findings underscore the overlapping roles of vasopressin and oxytocin signaling in ASD.

### 7.3. Dopamine

The functional role of dopamine in reward processing is well-established [250]. Based on the social motivation hypothesis, dopamine system dysfunction has been extensively investigated in ASD. This hypothesis posits that an impaired reward system is a key neurobiological substrate underlying reduced social interest in ASD [251].

Human neuroimaging studies have provided empirical support for this dysfunction. For instance, a positron emission tomographic study using radiolabeled fluorodopa showed that its binding is reduced by 39% in the anterior medial prefrontal cortex of individuals with ASD [252]. Furthermore, anatomical magnetic resonance imaging studies have demonstrated structural alterations of the dopamine system, including an enlarged caudate nucleus, and reduced grey matter volume in fronto-striatal networks [253,254]. Functional imaging has also consistently revealed increased striato-cortical connectivity in individuals with ASD, suggesting a disorganized communication within dopamine-mediated circuits (reviewed in [251]). Collectively, these findings underscore the significant involvement of dopaminergic impairment in the pathophysiology of ASD.

Genetic analyses have identified various ASD-associated polymorphisms in genes involved in dopamine signaling [255]. While several candidates have emerged, the *SLC6A3* gene (encoding the dopamine active transporter, DAT) stands out as one of the most consistently validated risk factors across independent studies [256,257]. Notably, a de novo mutation, T356M, was identified in *SLC6A3* in individuals with ASD [53,256]. Functional validation in mice harboring homozygous mutation of T356M demonstrated ASD-relevant phenotypes, including reduced sociability, highlighting the clinical significance of dopamine signaling [258].

### 7.4. Endocannabinoid System

The endocannabinoid system has recently gained significant attention for its involvement in ASD [259,260,261]. The endocannabinoid system regulates E/I balance by modulating synaptic transmission [262]. Cannabinoid receptors, located at presynaptic terminals, were originally identified as the target for Δ^9^-tetrahydrocannabinol [263], the primary psychoactive constituent of cannabis, and endogenous ligands are found later [264]. Endogenous ligands, such as 2-arachidonoylglycerol (2-AG) and anandamide (AEA), are released from postsynaptic neurons and bind to presynaptic receptors [262]. This retrograde signaling primarily attenuates synaptic strength through long-term depression [262].

While neuroimaging evidence investigating the endocannabinoid system in individuals with ASD remains scarce, multiple case–control studies have reported reduced blood anandamide levels in children with ASD [265,266]. They have been shown to correlate with the severity of social impairment [265]. These results suggest that anandamide can be used as a potential biomarker for the disorder.

Postmortem evidence is similarly limited. In a case–control study, a downregulation of cannabinoid receptor type 1 has been observed in brain tissues from individuals with ASD [267]. While further robust human data are urgently required, alterations in the endocannabinoid system have been reported across various ASD model mice [260].

The therapeutic potential of cannabinoids has been evaluated in clinical settings. In a recent double-blind randomized controlled trial, individuals with ASD were treated for 12 weeks with either a whole-plant cannabis extract with Δ^9^-tetrahydrocannabinol, purified cannabidiol with Δ^9^-tetrahydrocannabinol, or a placebo [268]. The cannabinoid treatment led to improvement in some behavioral metrics, including Clinical Global Impression-Improvement scale with disruptive behavior anchor points (CGI-I), and Social Responsiveness Scale (SRS-2). However, no significant differences were observed in other behavioral outcomes, yielding mixed results regarding the overall efficacy of these interventions.

## 8. Cerebellar Impairment

The cerebellum has traditionally been regarded as a center of motor coordination and learning. Over the past decades, accumulating evidence has expanded this view to include roles in higher cognitive and affective functions [269], prompting growing interest in its relevance to ASD [95]. Epidemiological and neuropathological findings support a potential association between cerebellar dysfunction and ASD. Perinatal cerebellar injury increases the risk of ASD 36-fold [270], and postmortem analyses consistently report reductions in Purkinje cell number in affected individuals [271,272]. The consistency of post-mortem observations and high-risk ratios underscores the cerebellar involvement in a non-negligible fraction of individuals with idiopathic ASD.

Anatomically, the cerebellum forms reciprocal connections with the neocortex through topographically organized cerebro-cerebellar loops that correspond to distinct cortical regions. Functional human imaging studies indicate that deficits in motor control and repetitive behaviors are associated with sensory-motor cerebro-cerebellar circuits, whereas deficits in social domains are linked to circuits that are involved in language and social cognition [273]. Abnormalities in the right cerebellar Crus I/II have been associated with greater symptom severity across ASD-relevant behavioral domains [273], and alterations of functional connectivity between cortical association areas and the right Crus I have been observed in individuals with ASD [274]. Nevertheless, the extent to which idiopathic ASD can be considered primarily cerebellar-dependent remains uncertain.

Animal models provide causal evidence that cerebellar perturbations can elicit ASD-relevant behaviors. The Purkinje cell-specific deletion of *Tsc1*, a gene that is mutated in tuberous sclerosis complex, induces impairments in sociability, repetitive behaviors, and inflexible learning [275]. The timed correction of mammalian target of rapamycin (mTOR) signaling rescues subsets of these phenotypes in a domain-specific manner, indicating distinct critical periods for cerebellar contributions to behavior [276].

Subsequent studies that selectively manipulated other ASD-associated genes in Purkinje cells further support cerebellar involvement [277,278,279,280]. The transient developmental dysfunction of Purkinje cells produces region-specific behavioral alterations. Chemogenetic activation during postnatal development (postnatal days 30–56) elicits lobule-specific impairments in social interaction, repetitive behaviors, and cognitive inflexibility, whereas acute activation in adulthood preferentially affects reversal learning [281]. Likewise, the acute optogenetic inhibition of adult Purkinje cells in the right Crus I induces all core ASD-like behavioral features, whereas chemogenetic activation in *Tsc1*-deleted mice rescues social impairments without significantly affecting repetitive behaviors or cognitive inflexibility [274].

Circuit-level analyses implicate cerebellar output pathways in these effects. In Purkinje cell-specific *Tsc1* deletion models, thalamocortical connectivity is altered [282]. These studies delineate a pathway that links the right Crus I and posterior vermis with the ventromedial thalamus and medial prefrontal cortex, forming a cerebello-thalamo-prefrontal circuit. The acute bidirectional manipulation of ventromedial thalamic projections to the medial prefrontal cortex modulates ASD-relevant behaviors, providing causal evidence of the involvement of this pathway.

Recent rodent work further demonstrates that the cerebellum provides extensive projections throughout the cerebral cortex [283,284] and that cerebellar outputs are organized in a modular manner [285]. Although cerebellar-targeted interventions in humans remain at an early stage, these findings suggest that the cerebellum represents a potentially important target for ameliorating ASD-related deficits.

## 9. Gene × Environment Interaction

We have previously reviewed the effects of genetic factors alone, environmental factors alone, and examples in which environmental exposures influence epigenetic regulation. However, as discussed in the environmental factors section, there are substantial methodological challenges in detecting gene–environment (G × E) interactions in practice [286,287].

By definition, a G × E interaction exists when the risk of ASD associated with a given environmental exposure differs according to genotype, such that individuals with a particular genetic variant show a significantly increased (or decreased) risk following exposure to a specific environmental factor. However, the effect sizes of environmental risk factors for ASD are generally modest, and only a small proportion of ASD cases in observational cohorts are expected to be attributable to the exposure of interest. In this context of substantial background noise, stratifying individuals by genotype and detecting interactions that require greater statistical power is highly challenging [286].

Furthermore, as the number of candidate genes increases, the number of testable G × E interactions grows combinatorially, raising concerns regarding multiple testing and false positives. In addition, publication bias toward positive findings may further distort the literature. Indeed, a study examining G × E research in psychiatric disorders published between 2000 and 2009 reported evidence of such bias [288]. Consequently, replicated gene–environment interactions in ASD remain limited.

Several reviews have summarized reported G × E interactions in ASD [289,290]. Although replication is limited, a number of candidate findings have been reported. A prototypical example of a proposed G × E interaction in ASD is the finding that the association between air pollution exposure and ASD risk may be modified by variation in the MET receptor tyrosine kinase (*MET*) gene [291]. In this case–control study, individuals homozygous for the C allele of the *MET* polymorphism (rs1858830) exhibited an increased risk of ASD under conditions of high exposure to air pollution. The C allele has been associated with reduced MET protein expression [292], and reduced MET signaling is hypothesized to increase vulnerability to environmental toxicants, although the underlying biological mechanisms remain incompletely understood.

Similarly, a trio study has revealed that polymorphisms associated with reduced activity of paraoxonase 1 (*PON1*), an enzyme involved in the detoxification of organophosphate pesticides, are associated with increased ASD risk in populations with high pesticide exposure in North America, but not in Italy [293]. Moreover, reduced PON1 arylesterase activity has been observed in individuals with ASD compared to the first-degree relatives and controls [294]. However, these findings have not been replicated in other case–control and family-based association studies [295,296].

Polymorphisms in glutathione S-transferase M1 (*GSTM1*), which is involved in detoxification of heavy metals and oxidative stress, have also been implicated. Trio-based analyses have shown an increased frequency of the *GSTM1*-null genotype in ASD cases [297], and another case–control study reported a non-significant trend in the same direction [298]. In addition, a family-based association study revealed that a maternal *GSTP1* polymorphism has been associated with a 2.7-fold increased risk of having a child with ASD [299].

Taken together, these findings suggest that genetic variants may contribute to ASD risk by modulating cellular toxic burden in response to environmental exposures. However, further studies are required to elucidate the biological mechanisms by which such variants modify responses to environmental factors and contribute to ASD pathogenesis. Moreover, given methodological limitations and susceptibility to false-positive findings, replication using multiple complementary study designs is essential to validate reported interactions.

## 10. Summary

Despite significant advances in human and animal research, the etiology of ASD remains incompletely understood, with most cases still classified as idiopathic. Genetic factors play a central role, interacting with environmental influences throughout development to generate highly heterogeneous neurobiological and behavioral phenotypes. Variability in penetrance, sex bias, and symptom severity remain important unresolved issues. Addressing these issues is essential for integrating diverse biological findings into a coherent understanding of the pathogenesis of ASD and guiding the development of effective, targeted interventions.

Organizing diverse risk factors along a developmental timeline may provide a useful conceptual framework for integrating inherited genetic liability, prenatal and perinatal perturbations, and neurodevelopmental processes, as schematically illustrated in Figure 1. This perspective may help explain why diverse clinical features arise from shared biological mechanisms and may also inform future mechanistic and translational studies.

## Figures and Tables

**Table 1:** Odds ratio and prevalence of recurrent CNVs.

CNV	Odds Ratio	Prevalence in Cases (%)	Notes and References
1q21.1 del	5.7	1.2	[27]
1q21.1 dup	5.3	1.0	[27]
7q11.23 del	16.0	0.7	Williams syndrome (increased sociability) [28]
7q11.23 dup	7.1	0.5	[28]
15q11-13.1 del *	2.5	0.7	[29]
15q11-13.1 dup *	4.0	1.4	[29]
15q13.3 del	3.6	1.3	[30]
15q13.3 dup	4.2	0.5	[31]
16p11.2 del **	8.3	2.3	[32]
16p11.2 dup	3.2	1.4	[32]
22q11.21 del	16.7	2.3	DiGeorge syndrome [33]
22q11.21 dup	2.5	1.0	[34]
22q11.22 del	3.3	0.5	
22q11.22 dup	Infinity	0.2	

* These loci are responsible for Prader–Willi and Angelman syndromes. ** Two loci are combined.

**Table 2:** Representative syndromic forms of ASD.

Syndrome	Gene	Mutation Type	ASD Prevalence	Key Clinical Features
Fragile X Syndrome	*FMR1*	CGG repeat expansion	50% in males	Facial abnormalities Intellectual disability [64]
Phelan-McDermid Syndrome	*SHANK3*	Loss of function	60–94%	Intellectual disability Hypotonia [65]
Tuberous Sclerosis Complex	*TSC1*, *TSC2*	Loss of function	26–45%	Epilepsy Multiple hamartomas [66]
Cornelia de Lange Syndrome	*NIPBL*, *SMC1*	Loss of function	43%	Facial abnormalities Intellectual disability [67]
Timothy Syndrome	*CACNA1C*	Gain of function	44–50%	Long QT syndrome Syndactyly [68]

## Data Availability

No new data were created or analyzed in this study. Data sharing is not applicable to this article.

## Abbreviations

The following abbreviations are used in this manuscript:

ASD	autism spectrum disorder
CNV	copy number variant
del	deletion
dup	duplication
E/I	excitation/inhibition
GABA	γ-aminobutyric acid
GAD	glutamate decarboxylase
HDAC	histone deacetylase
IL	Interleukin
kb	Kilobases
OR	odds ratio
Poly(I:C)	polyinosinic-polycytidylic acid
SFARI	Simons Foundation Autism Research Initiative
SNV	single-nucleotide variant
SSRI	selective serotonin reuptake inhibitor
VPA	valproic acid

## References

[1] Tye C., Runicles A.K., Whitehouse A.J.O., Alvares G.A. (2018). Characterizing the Interplay Between Autism Spectrum Disorder and Comorbid Medical Conditions: An Integrative Review. Front. Psychiatry.

[2] Zeidan J., Fombonne E., Scorah J., Ibrahim A., Durkin M.S., Saxena S., Yusuf A., Shih A., Elsabbagh M. (2022). Global prevalence of autism: A systematic review update. Autism Res..

[3] Bourgeron T. (2016). Current knowledge on the genetics of autism and propositions for future research. Comptes Rendus Biol..

[4] Zablotsky B., Black L.I., Maenner M.J., Schieve L.A., Blumberg S.J. (2015). Estimated Prevalence of Autism and Other Developmental Disabilities Following Questionnaire Changes in the 2014 National Health Interview Survey. Natl. Health Stat. Rep..

[5] Talantseva O.I., Romanova R.S., Shurdova E.M., Dolgorukova T.A., Sologub P.S., Titova O.S., Kleeva D.F., Grigorenko E.L. (2023). The global prevalence of autism spectrum disorder: A three-level meta-analysis. Front. Psychiatry.

[6] Santomauro D.F., Erskine H.E., Herrera A.M.M., Miller P.A., Shadid J., Hagins H., Addo I.Y., Adnani Q.E.S., Ahinkorah B.O., Ahmed A. (2025). The global epidemiology and health burden of the autism spectrum: Findings from the Global Burden of Disease Study 2021. Lancet Psychiatry.

[7] Shaw K.A., Williams S., Patrick M.E., Valencia-Prado M., Durkin M.S., Howerton E.M., Ladd-Acosta C.M., Pas E.T., Bakian A.V., Bartholomew P. (2025). Prevalence and Early Identification of Autism Spectrum Disorder Among Children Aged 4 and 8 Years—Autism and Developmental Disabilities Monitoring Network, 16 Sites, United States, 2022. MMWR Surveill. Summ..

[8] Beggiato A., Peyre H., Maruani A., Scheid I., Rastam M., Amsellem F., Gillberg C.I., Leboyer M., Bourgeron T., Gillberg C. (2017). Gender differences in autism spectrum disorders: Divergence among specific core symptoms. Autism Res..

[9] Tick B., Bolton P., Happé F., Rutter M., Rijsdijk F. (2016). Heritability of autism spectrum disorders: A meta-analysis of twin studies. J. Child. Psychol. Psychiatry.

[10] Sandin S., Lichtenstein P., Kuja-Halkola R., Larsson H., Hultman C.M., Reichenberg A. (2014). The familial risk of autism. JAMA.

[11] Losh M., Childress D., Lam K., Piven J. (2008). Defining key features of the broad autism phenotype: A comparison across parents of multiple- and single-incidence autism families. Am. J. Med. Genet. B Neuropsychiatr. Genet..

[12] Ozonoff S., Young G.S., Bradshaw J., Charman T., Chawarska K., Iverson J.M., Klaiman C., Landa R.J., McDonald N., Messinger D. (2024). Familial Recurrence of Autism: Updates from the Baby Siblings Research Consortium. Pediatrics.

[13] Geschwind D.H., State M.W. (2015). Gene hunting in autism spectrum disorder: On the path to precision medicine. Lancet Neurol..

[14] Trost B., Thiruvahindrapuram B., Chan A.J.S., Engchuan W., Higginbotham E.J., Howe J.L., Loureiro L.O., Reuter M.S., Roshandel D., Whitney J. (2022). Genomic architecture of autism from comprehensive whole-genome sequence annotation. Cell.

[15] Abrahams B.S., Arking D.E., Campbell D.B., Mefford H.C., Morrow E.M., Weiss L.A., Menashe I., Wadkins T., Banerjee-Basu S., Packer A. (2013). SFARI Gene 2.0: A community-driven knowledgebase for the autism spectrum disorders (ASDs). Mol. Autism.

[16] Malhotra D., Sebat J. (2012). CNVs: Harbingers of a rare variant revolution in psychiatric genetics. Cell.

[17] Robinson W.P. (2000). Mechanisms leading to uniparental disomy and their clinical consequences. Bioessays.

[18] Zhang F., Gu W., Hurles M.E., Lupski J.R. (2009). Copy number variation in human health, disease, and evolution. Annu. Rev. Genom. Hum. Genet..

[19] Itsara A., Wu H., Smith J.D., Nickerson D.A., Romieu I., London S.J., Eichler E.E. (2010). De novo rates and selection of large copy number variation. Genome Res..

[20] Abedini S.S., Akhavantabasi S., Liang Y., Heng J.I., Alizadehsani R., Dehzangi I., Bauer D.C., Alinejad-Rokny H. (2024). A critical review of the impact of candidate copy number variants on autism spectrum disorder. Mutat. Res. Rev. Mutat. Res..

[21] Marshall C.R., Noor A., Vincent J.B., Lionel A.C., Feuk L., Skaug J., Shago M., Moessner R., Pinto D., Ren Y. (2008). Structural variation of chromosomes in autism spectrum disorder. Am. J. Hum. Genet..

[22] Pinto D., Pagnamenta A.T., Klei L., Anney R., Merico D., Regan R., Conroy J., Magalhaes T.R., Correia C., Abrahams B.S. (2010). Functional impact of global rare copy number variation in autism spectrum disorders. Nature.

[23] Sanders S.J., Ercan-Sencicek A.G., Hus V., Luo R., Murtha M.T., Moreno-De-Luca D., Chu S.H., Moreau M.P., Gupta A.R., Thomson S.A. (2011). Multiple recurrent de novo CNVs, including duplications of the 7q11.23 Williams syndrome region, are strongly associated with autism. Neuron.

[24] Tamada K., Takumi T. (2025). Neurodevelopmental impact of CNV models in ASD: Recent advances and future directions. Curr. Opin. Neurobiol..

[25] Willsey H.R., Willsey A.J., Wang B., State M.W. (2022). Genomics, convergent neuroscience and progress in understanding autism spectrum disorder. Nat. Rev. Neurosci..

[26] Willsey A.J., Sanders S.J., Li M., Dong S., Tebbenkamp A.T., Muhle R.A., Reilly S.K., Lin L., Fertuzinhos S., Miller J.A. (2013). Coexpression networks implicate human midfetal deep cortical projection neurons in the pathogenesis of autism. Cell.

[27] Bernier R., Steinman K.J., Reilly B., Wallace A.S., Sherr E.H., Pojman N., Mefford H.C., Gerdts J., Earl R., Hanson E. (2016). Clinical phenotype of the recurrent 1q21.1 copy-number variant. Genet. Med..

[28] López-Tobón A., Trattaro S., Testa G. (2020). The sociability spectrum: Evidence from reciprocal genetic copy number variations. Mol. Autism.

[29] Veltman M.W., Craig E.E., Bolton P.F. (2005). Autism spectrum disorders in Prader-Willi and Angelman syndromes: A systematic review. Psychiatr. Genet..

[30] Deutsch S.I., Burket J.A., Benson A.D., Urbano M.R. (2016). The 15q13.3 deletion syndrome: Deficient α(7)-containing nicotinic acetylcholine receptor-mediated neurotransmission in the pathogenesis of neurodevelopmental disorders. Prog. Neuropsychopharmacol. Biol. Psychiatry.

[31] Genovese A., Butler M.G. (2023). The Autism Spectrum: Behavioral, Psychiatric and Genetic Associations. Genes.

[32] Rein B., Yan Z. (2020). 16p11.2 Copy Number Variations and Neurodevelopmental Disorders. Trends Neurosci..

[33] Zinkstok J.R., Boot E., Bassett A.S., Hiroi N., Butcher N.J., Vingerhoets C., Vorstman J.A.S., van Amelsvoort T. (2019). Neurobiological perspective of 22q11.2 deletion syndrome. Lancet Psychiatry.

[34] Wenger T.L., Miller J.S., DePolo L.M., de Marchena A.B., Clements C.C., Emanuel B.S., Zackai E.H., McDonald-McGinn D.M., Schultz R.T. (2016). 22q11.2 duplication syndrome: Elevated rate of autism spectrum disorder and need for medical screening. Mol. Autism.

[35] Sebat J., Lakshmi B., Malhotra D., Troge J., Lese-Martin C., Walsh T., Yamrom B., Yoon S., Krasnitz A., Kendall J. (2007). Strong association of de novo copy number mutations with autism. Science.

[36] Lupski J.R. (2007). Genomic rearrangements and sporadic disease. Nat. Genet..

[37] Hehir-Kwa J.Y., Rodríguez-Santiago B., Vissers L.E., de Leeuw N., Pfundt R., Buitelaar J.K., Pérez-Jurado L.A., Veltman J.A. (2011). De novo copy number variants associated with intellectual disability have a paternal origin and age bias. J. Med. Genet..

[38] Wadhawan I., Hai Y., Foyouzi Yousefi N., Guo X., Graham J.M., Rosenfeld J.A. (2020). De novo copy number variants and parental age: Is there an association?. Eur. J. Med. Genet..

[39] Belyeu J.R., Brand H., Wang H., Zhao X., Pedersen B.S., Feusier J., Gupta M., Nicholas T.J., Brown J., Baird L. (2021). De novo structural mutation rates and gamete-of-origin biases revealed through genome sequencing of 2396 families. Am. J. Hum. Genet..

[40] Duyzend M.H., Nuttle X., Coe B.P., Baker C., Nickerson D.A., Bernier R., Eichler E.E. (2016). Maternal Modifiers and Parent-of-Origin Bias of the Autism-Associated 16p11.2 CNV. Am. J. Hum. Genet..

[41] Sanders S.J., He X., Willsey A.J., Ercan-Sencicek A.G., Samocha K.E., Cicek A.E., Murtha M.T., Bal V.H., Bishop S.L., Dong S. (2015). Insights into Autism Spectrum Disorder Genomic Architecture and Biology from 71 Risk Loci. Neuron.

[42] Girirajan S., Campbell C.D., Eichler E.E. (2011). Human copy number variation and complex genetic disease. Annu. Rev. Genet..

[43] Nakatani J., Tamada K., Hatanaka F., Ise S., Ohta H., Inoue K., Tomonaga S., Watanabe Y., Chung Y.J., Banerjee R. (2009). Abnormal behavior in a chromosome-engineered mouse model for human 15q11-13 duplication seen in autism. Cell.

[44] Nakai N., Nagano M., Saitow F., Watanabe Y., Kawamura Y., Kawamoto A., Tamada K., Mizuma H., Onoe H., Monai H. (2017). Serotonin rebalances cortical tuning and behavior linked to autism symptoms in 15q11-13 CNV mice. Sci. Adv..

[45] Tamada K., Fukumoto K., Toya T., Nakai N., Awasthi J.R., Tanaka S., Okabe S., Spitz F., Saitow F., Suzuki H. (2021). Genetic dissection identifies Necdin as a driver gene in a mouse model of paternal 15q duplications. Nat. Commun..

[46] Albrecht U., Sutcliffe J.S., Cattanach B.M., Beechey C.V., Armstrong D., Eichele G., Beaudet A.L. (1997). Imprinted expression of the murine Angelman syndrome gene, *Ube3a*, in hippocampal and Purkinje neurons. Nat. Genet..

[47] Smith S.E., Zhou Y.D., Zhang G., Jin Z., Stoppel D.C., Anderson M.P. (2011). Increased gene dosage of *Ube3a* results in autism traits and decreased glutamate synaptic transmission in mice. Sci. Transl. Med..

[48] Horev G., Ellegood J., Lerch J.P., Son Y.E., Muthuswamy L., Vogel H., Krieger A.M., Buja A., Henkelman R.M., Wigler M. (2011). Dosage-dependent phenotypes in models of 16p11.2 lesions found in autism. Proc. Natl. Acad. Sci. USA.

[49] Forrest M.P., Dos Santos M., Piguel N.H., Wang Y.Z., Hawkins N.A., Bagchi V.A., Dionisio L.E., Yoon S., Simkin D., Martin-de-Saavedra M.D. (2023). Rescue of neuropsychiatric phenotypes in a mouse model of 16p11.2 duplication syndrome by genetic correction of an epilepsy network hub. Nat. Commun..

[50] O’Roak B.J., Deriziotis P., Lee C., Vives L., Schwartz J.J., Girirajan S., Karakoc E., Mackenzie A.P., Ng S.B., Baker C. (2011). Exome sequencing in sporadic autism spectrum disorders identifies severe de novo mutations. Nat. Genet..

[51] O’Roak B.J., Vives L., Girirajan S., Karakoc E., Krumm N., Coe B.P., Levy R., Ko A., Lee C., Smith J.D. (2012). Sporadic autism exomes reveal a highly interconnected protein network of de novo mutations. Nature.

[52] De Rubeis S., He X., Goldberg A.P., Poultney C.S., Samocha K., Cicek A.E., Kou Y., Liu L., Fromer M., Walker S. (2014). Synaptic, transcriptional and chromatin genes disrupted in autism. Nature.

[53] Neale B.M., Kou Y., Liu L., Ma’ayan A., Samocha K.E., Sabo A., Lin C.F., Stevens C., Wang L.S., Makarov V. (2012). Patterns and rates of exonic de novo mutations in autism spectrum disorders. Nature.

[54] Iossifov I., O’Roak B.J., Sanders S.J., Ronemus M., Krumm N., Levy D., Stessman H.A., Witherspoon K.T., Vives L., Patterson K.E. (2014). The contribution of de novo coding mutations to autism spectrum disorder. Nature.

[55] Sanders S.J., Murtha M.T., Gupta A.R., Murdoch J.D., Raubeson M.J., Willsey A.J., Ercan-Sencicek A.G., DiLullo N.M., Parikshak N.N., Stein J.L. (2012). De novo mutations revealed by whole-exome sequencing are strongly associated with autism. Nature.

[56] Belmadani M., Jacobson M., Holmes N., Phan M., Nguyen T., Pavlidis P., Rogic S. (2019). VariCarta: A Comprehensive Database of Harmonized Genomic Variants Found in Autism Spectrum Disorder Sequencing Studies. Autism Res..

[57] Porubsky D., Dashnow H., Sasani T.A., Logsdon G.A., Hallast P., Noyes M.D., Kronenberg Z.N., Mokveld T., Koundinya N., Nolan C. (2025). Human de novo mutation rates from a four-generation pedigree reference. Nature.

[58] Satterstrom F.K., Kosmicki J.A., Wang J., Breen M.S., De Rubeis S., An J.Y., Peng M., Collins R., Grove J., Klei L. (2020). Large-Scale Exome Sequencing Study Implicates Both Developmental and Functional Changes in the Neurobiology of Autism. Cell.

[59] An J.Y., Lin K., Zhu L., Werling D.M., Dong S., Brand H., Wang H.Z., Zhao X., Schwartz G.B., Collins R.L. (2018). Genome-wide de novo risk score implicates promoter variation in autism spectrum disorder. Science.

[60] Kong A., Frigge M.L., Masson G., Besenbacher S., Sulem P., Magnusson G., Gudjonsson S.A., Sigurdsson A., Jonasdottir A., Wong W.S. (2012). Rate of de novo mutations and the importance of father’s age to disease risk. Nature.

[61] Jónsson H., Sulem P., Kehr B., Kristmundsdottir S., Zink F., Hjartarson E., Hardarson M.T., Hjorleifsson K.E., Eggertsson H.P., Gudjonsson S.A. (2017). Parental influence on human germline de novo mutations in 1548 trios from Iceland. Nature.

[62] Sandin S., Schendel D., Magnusson P., Hultman C., Surén P., Susser E., Grønborg T., Gissler M., Gunnes N., Gross R. (2016). Autism risk associated with parental age and with increasing difference in age between the parents. Mol. Psychiatry.

[63] Aitken R.J., De Iuliis G.N., Nixon B. (2020). The Sins of Our Forefathers: Paternal Impacts on De Novo Mutation Rate and Development. Annu. Rev. Genet..

[64] Hagerman R.J., Berry-Kravis E., Hazlett H.C., Bailey D.B., Moine H., Kooy R.F., Tassone F., Gantois I., Sonenberg N., Mandel J.L. (2017). Fragile X syndrome. Nat. Rev. Dis. Primers.

[65] Soorya L., Kolevzon A., Zweifach J., Lim T., Dobry Y., Schwartz L., Frank Y., Wang A.T., Cai G., Parkhomenko E. (2013). Prospective investigation of autism and genotype-phenotype correlations in 22q13 deletion syndrome and *SHANK3* deficiency. Mol. Autism.

[66] Specchio N., Pietrafusa N., Trivisano M., Moavero R., De Palma L., Ferretti A., Vigevano F., Curatolo P. (2020). Autism and Epilepsy in Patients with Tuberous Sclerosis Complex. Front. Neurol..

[67] Richards C., Jones C., Groves L., Moss J., Oliver C. (2015). Prevalence of autism spectrum disorder phenomenology in genetic disorders: A systematic review and meta-analysis. Lancet Psychiatry.

[68] Marcantoni A., Calorio C., Hidisoglu E., Chiantia G., Carbone E. (2020). Cav1.2 channelopathies causing autism: New hallmarks on Timothy syndrome. Pflug. Arch..

[69] Krumm N., Turner T.N., Baker C., Vives L., Mohajeri K., Witherspoon K., Raja A., Coe B.P., Stessman H.A., He Z.X. (2015). Excess of rare, inherited truncating mutations in autism. Nat. Genet..

[70] Cirnigliaro M., Chang T.S., Arteaga S.A., Pérez-Cano L., Ruzzo E.K., Gordon A., Bicks L.K., Jung J.Y., Lowe J.K., Wall D.P. (2023). The contributions of rare inherited and polygenic risk to ASD in multiplex families. Proc. Natl. Acad. Sci. USA.

[71] The Wellcome Trust Case Control Consortium (2007). Genome-wide association study of 14,000 cases of seven common diseases and 3000 shared controls. Nature.

[72] Grove J., Ripke S., Als T.D., Mattheisen M., Walters R.K., Won H., Pallesen J., Agerbo E., Andreassen O.A., Anney R. (2019). Identification of common genetic risk variants for autism spectrum disorder. Nat. Genet..

[73] Gaugler T., Klei L., Sanders S.J., Bodea C.A., Goldberg A.P., Lee A.B., Mahajan M., Manaa D., Pawitan Y., Reichert J. (2014). Most genetic risk for autism resides with common variation. Nat. Genet..

[74] Kerin T., Ramanathan A., Rivas K., Grepo N., Coetzee G.A., Campbell D.B. (2012). A noncoding RNA antisense to moesin at 5p14.1 in autism. Sci. Transl. Med..

[75] Pinto D., Delaby E., Merico D., Barbosa M., Merikangas A., Klei L., Thiruvahindrapuram B., Xu X., Ziman R., Wang Z. (2014). Convergence of genes and cellular pathways dysregulated in autism spectrum disorders. Am. J. Hum. Genet..

[76] Parikshak N.N., Luo R., Zhang A., Won H., Lowe J.K., Chandran V., Horvath S., Geschwind D.H. (2013). Integrative functional genomic analyses implicate specific molecular pathways and circuits in autism. Cell.

[77] Velmeshev D., Schirmer L., Jung D., Haeussler M., Perez Y., Mayer S., Bhaduri A., Goyal N., Rowitch D.H., Kriegstein A.R. (2019). Single-cell genomics identifies cell type-specific molecular changes in autism. Science.

[78] Wamsley B., Bicks L., Cheng Y., Kawaguchi R., Quintero D., Margolis M., Grundman J., Liu J., Xiao S., Hawken N. (2024). Molecular cascades and cell type-specific signatures in ASD revealed by single-cell genomics. Science.

[79] Wong C.C., Meaburn E.L., Ronald A., Price T.S., Jeffries A.R., Schalkwyk L.C., Plomin R., Mill J. (2014). Methylomic analysis of monozygotic twins discordant for autism spectrum disorder and related behavioural traits. Mol. Psychiatry.

[80] Voineagu I., Wang X., Johnston P., Lowe J.K., Tian Y., Horvath S., Mill J., Cantor R.M., Blencowe B.J., Geschwind D.H. (2011). Transcriptomic analysis of autistic brain reveals convergent molecular pathology. Nature.

[81] Gupta S., Ellis S.E., Ashar F.N., Moes A., Bader J.S., Zhan J., West A.B., Arking D.E. (2014). Transcriptome analysis reveals dysregulation of innate immune response genes and neuronal activity-dependent genes in autism. Nat. Commun..

[82] Wong C.C.Y., Smith R.G., Hannon E., Ramaswami G., Parikshak N.N., Assary E., Troakes C., Poschmann J., Schalkwyk L.C., Sun W. (2019). Genome-wide DNA methylation profiling identifies convergent molecular signatures associated with idiopathic and syndromic autism in post-mortem human brain tissue. Hum. Mol. Genet..

[83] Gholamalizadeh H., Amiri-Shahri M., Rasouli F., Ansari A., Baradaran Rahimi V., Reza Askari V. (2024). DNA Methylation in Autism Spectrum Disorders: Biomarker or Pharmacological Target?. Brain Sci..

[84] Amir R.E., Van den Veyver I.B., Wan M., Tran C.Q., Francke U., Zoghbi H.Y. (1999). Rett syndrome is caused by mutations in X-linked *MECP2*, encoding methyl-CpG-binding protein 2. Nat. Genet..

[85] Sandweiss A.J., Brandt V.L., Zoghbi H.Y. (2020). Advances in understanding of Rett syndrome and *MECP2* duplication syndrome: Prospects for future therapies. Lancet Neurol..

[86] Tsujimura K., Irie K., Nakashima H., Egashira Y., Fukao Y., Fujiwara M., Itoh M., Uesaka M., Imamura T., Nakahata Y. (2015). miR-199a Links MeCP2 with mTOR Signaling and Its Dysregulation Leads to Rett Syndrome Phenotypes. Cell Rep..

[87] Gabel H.W., Kinde B., Stroud H., Gilbert C.S., Harmin D.A., Kastan N.R., Hemberg M., Ebert D.H., Greenberg M.E. (2015). Disruption of DNA-methylation-dependent long gene repression in Rett syndrome. Nature.

[88] Bernier R., Golzio C., Xiong B., Stessman H.A., Coe B.P., Penn O., Witherspoon K., Gerdts J., Baker C., Vulto-van Silfhout A.T. (2014). Disruptive *CHD8* mutations define a subtype of autism early in development. Cell.

[89] Kleefstra T., Smidt M., Banning M.J., Oudakker A.R., Van Esch H., de Brouwer A.P., Nillesen W., Sistermans E.A., Hamel B.C., de Bruijn D. (2005). Disruption of the gene *Euchromatin Histone Methyl Transferase1* (*Eu-HMTase1*) is associated with the 9q34 subtelomeric deletion syndrome. J. Med. Genet..

[90] Jamain S., Quach H., Betancur C., Råstam M., Colineaux C., Gillberg I.C., Soderstrom H., Giros B., Leboyer M., Gillberg C. (2003). Mutations of the X-linked genes encoding neuroligins NLGN3 and NLGN4 are associated with autism. Nat. Genet..

[91] Tabuchi K., Blundell J., Etherton M.R., Hammer R.E., Liu X., Powell C.M., Südhof T.C. (2007). A neuroligin-3 mutation implicated in autism increases inhibitory synaptic transmission in mice. Science.

[92] Kazdoba T.M., Leach P.T., Crawley J.N. (2016). Behavioral phenotypes of genetic mouse models of autism. Genes Brain Behav..

[93] Hisle-Gorman E., Susi A., Stokes T., Gorman G., Erdie-Lalena C., Nylund C.M. (2018). Prenatal, perinatal, and neonatal risk factors of autism spectrum disorder. Pediatr. Res..

[94] Sato A., Kotajima-Murakami H., Tanaka M., Katoh Y., Ikeda K. (2022). Influence of Prenatal Drug Exposure, Maternal Inflammation, and Parental Aging on the Development of Autism Spectrum Disorder. Front. Psychiatry.

[95] Wang S.S., Kloth A.D., Badura A. (2014). The cerebellum, sensitive periods, and autism. Neuron.

[96] Modabbernia A., Velthorst E., Reichenberg A. (2017). Environmental risk factors for autism: An evidence-based review of systematic reviews and meta-analyses. Mol. Autism.

[97] Tordjman S., Somogyi E., Coulon N., Kermarrec S., Cohen D., Bronsard G., Bonnot O., Weismann-Arcache C., Botbol M., Lauth B. (2014). Gene × Environment interactions in autism spectrum disorders: Role of epigenetic mechanisms. Front. Psychiatry.

[98] Kim Y.S., Leventhal B.L. (2015). Genetic epidemiology and insights into interactive genetic and environmental effects in autism spectrum disorders. Biol. Psychiatry.

[99] Estes M.L., McAllister A.K. (2016). Maternal immune activation: Implications for neuropsychiatric disorders. Science.

[100] Pape K., Tamouza R., Leboyer M., Zipp F. (2019). Immunoneuropsychiatry—Novel perspectives on brain disorders. Nat. Rev. Neurol..

[101] Mednick S.A., Machon R.A., Huttunen M.O., Bonett D. (1988). Adult schizophrenia following prenatal exposure to an influenza epidemic. Arch. Gen. Psychiatry.

[102] Atladóttir H.O., Thorsen P., Østergaard L., Schendel D.E., Lemcke S., Abdallah M., Parner E.T. (2010). Maternal infection requiring hospitalization during pregnancy and autism spectrum disorders. J. Autism Dev. Disord..

[103] Atladóttir H.Ó., Henriksen T.B., Schendel D.E., Parner E.T. (2012). Autism after infection, febrile episodes, and antibiotic use during pregnancy: An exploratory study. Pediatrics.

[104] Jiang H.Y., Xu L.L., Shao L., Xia R.M., Yu Z.H., Ling Z.X., Yang F., Deng M., Ruan B. (2016). Maternal infection during pregnancy and risk of autism spectrum disorders: A systematic review and meta-analysis. Brain Behav. Immun..

[105] Tioleco N., Silberman A.E., Stratigos K., Banerjee-Basu S., Spann M.N., Whitaker A.H., Turner J.B. (2021). Prenatal maternal infection and risk for autism in offspring: A meta-analysis. Autism Res..

[106] Goines P.E., Croen L.A., Braunschweig D., Yoshida C.K., Grether J., Hansen R., Kharrazi M., Ashwood P., Van de Water J. (2011). Increased midgestational IFN-γ, IL-4 and IL-5 in women bearing a child with autism: A case-control study. Mol. Autism.

[107] He H., Yu Y., Liew Z., Gissler M., László K.D., Valdimarsdóttir U.A., Zhang J., Li F., Li J. (2022). Association of Maternal Autoimmune Diseases with Risk of Mental Disorders in Offspring in Denmark. JAMA Netw. Open.

[108] Chen S.W., Zhong X.S., Jiang L.N., Zheng X.Y., Xiong Y.Q., Ma S.J., Qiu M., Huo S.T., Ge J., Chen Q. (2016). Maternal autoimmune diseases and the risk of autism spectrum disorders in offspring: A systematic review and meta-analysis. Behav. Brain Res..

[109] Brimberg L., Sadiq A., Gregersen P.K., Diamond B. (2013). Brain-reactive IgG correlates with autoimmunity in mothers of a child with an autism spectrum disorder. Mol. Psychiatry.

[110] Ashwood P., Krakowiak P., Hertz-Picciotto I., Hansen R., Pessah I.N., Van de Water J. (2011). Associations of impaired behaviors with elevated plasma chemokines in autism spectrum disorders. J. Neuroimmunol..

[111] Ashwood P., Krakowiak P., Hertz-Picciotto I., Hansen R., Pessah I., Van de Water J. (2011). Elevated plasma cytokines in autism spectrum disorders provide evidence of immune dysfunction and are associated with impaired behavioral outcome. Brain Behav. Immun..

[112] Saghazadeh A., Ataeinia B., Keynejad K., Abdolalizadeh A., Hirbod-Mobarakeh A., Rezaei N. (2019). A meta-analysis of pro-inflammatory cytokines in autism spectrum disorders: Effects of age, gender, and latitude. J. Psychiatr. Res..

[113] Vargas D.L., Nascimbene C., Krishnan C., Zimmerman A.W., Pardo C.A. (2005). Neuroglial activation and neuroinflammation in the brain of patients with autism. Ann. Neurol..

[114] Zimmerman A.W., Connors S.L., Matteson K.J., Lee L.C., Singer H.S., Castaneda J.A., Pearce D.A. (2007). Maternal antibrain antibodies in autism. Brain Behav. Immun..

[115] Wills S., Cabanlit M., Bennett J., Ashwood P., Amaral D.G., Van de Water J. (2009). Detection of autoantibodies to neural cells of the cerebellum in the plasma of subjects with autism spectrum disorders. Brain Behav. Immun..

[116] Goines P., Haapanen L., Boyce R., Duncanson P., Braunschweig D., Delwiche L., Hansen R., Hertz-Picciotto I., Ashwood P., Van de Water J. (2011). Autoantibodies to cerebellum in children with autism associate with behavior. Brain Behav. Immun..

[117] Solek C.M., Farooqi N., Verly M., Lim T.K., Ruthazer E.S. (2018). Maternal immune activation in neurodevelopmental disorders. Dev. Dyn..

[118] Shi L., Fatemi S.H., Sidwell R.W., Patterson P.H. (2003). Maternal influenza infection causes marked behavioral and pharmacological changes in the offspring. J. Neurosci..

[119] Fortier M.E., Joober R., Luheshi G.N., Boksa P. (2004). Maternal exposure to bacterial endotoxin during pregnancy enhances amphetamine-induced locomotion and startle responses in adult rat offspring. J. Psychiatr. Res..

[120] Meyer U., Nyffeler M., Engler A., Urwyler A., Schedlowski M., Knuesel I., Yee B.K., Feldon J. (2006). The time of prenatal immune challenge determines the specificity of inflammation-mediated brain and behavioral pathology. J. Neurosci..

[121] Smith S.E., Li J., Garbett K., Mirnics K., Patterson P.H. (2007). Maternal immune activation alters fetal brain development through interleukin-6. J. Neurosci..

[122] Santana-Coelho D., Layne-Colon D., Valdespino R., Ross C.C., Tardif S.D., O’Connor J.C. (2021). Advancing Autism Research from Mice to Marmosets: Behavioral Development of Offspring Following Prenatal Maternal Immune Activation. Front. Psychiatry.

[123] Choi G.B., Yim Y.S., Wong H., Kim S., Kim H., Kim S.V., Hoeffer C.A., Littman D.R., Huh J.R. (2016). The maternal interleukin-17a pathway in mice promotes autism-like phenotypes in offspring. Science.

[124] Garay P.A., Hsiao E.Y., Patterson P.H., McAllister A.K. (2013). Maternal immune activation causes age- and region-specific changes in brain cytokines in offspring throughout development. Brain Behav. Immun..

[125] Hsiao E.Y., McBride S.W., Chow J., Mazmanian S.K., Patterson P.H. (2012). Modeling an autism risk factor in mice leads to permanent immune dysregulation. Proc. Natl. Acad. Sci. USA.

[126] Mattei D., Ivanov A., Ferrai C., Jordan P., Guneykaya D., Buonfiglioli A., Schaafsma W., Przanowski P., Deuther-Conrad W., Brust P. (2017). Maternal immune activation results in complex microglial transcriptome signature in the adult offspring that is reversed by minocycline treatment. Transl. Psychiatry.

[127] Singer H.S., Morris C., Gause C., Pollard M., Zimmerman A.W., Pletnikov M. (2009). Prenatal exposure to antibodies from mothers of children with autism produces neurobehavioral alterations: A pregnant dam mouse model. J. Neuroimmunol..

[128] Martin L.A., Ashwood P., Braunschweig D., Cabanlit M., Van de Water J., Amaral D.G. (2008). Stereotypies and hyperactivity in rhesus monkeys exposed to IgG from mothers of children with autism. Brain Behav. Immun..

[129] Bauman M.D., Iosif A.M., Ashwood P., Braunschweig D., Lee A., Schumann C.M., Van de Water J., Amaral D.G. (2013). Maternal antibodies from mothers of children with autism alter brain growth and social behavior development in the rhesus monkey. Transl. Psychiatry.

[130] Brimberg L., Mader S., Jeganathan V., Berlin R., Coleman T.R., Gregersen P.K., Huerta P.T., Volpe B.T., Diamond B. (2016). Caspr2-reactive antibody cloned from a mother of an ASD child mediates an ASD-like phenotype in mice. Mol. Psychiatry.

[131] Agrawal S., Rao S.C., Bulsara M.K., Patole S.K. (2018). Prevalence of Autism Spectrum Disorder in Preterm Infants: A Meta-analysis. Pediatrics.

[132] Crump C., Sundquist J., Sundquist K. (2021). Preterm or Early Term Birth and Risk of Autism. Pediatrics.

[133] Persson M., Opdahl S., Risnes K., Gross R., Kajantie E., Reichenberg A., Gissler M., Sandin S. (2020). Gestational age and the risk of autism spectrum disorder in Sweden, Finland, and Norway: A cohort study. PLoS Med..

[134] Chang Y.S., Chen L.W., Yu T., Lin S.H., Kuo P.L. (2023). Preterm birth and weight-for-gestational age for risks of autism spectrum disorder and intellectual disability: A nationwide population-based cohort study. J. Formos. Med. Assoc..

[135] Rimol L.M., Botellero V.L., Bjuland K.J., Løhaugen G.C.C., Lydersen S., Evensen K.A.I., Brubakk A.M., Eikenes L., Indredavik M.S., Martinussen M. (2019). Reduced white matter fractional anisotropy mediates cortical thickening in adults born preterm with very low birthweight. Neuroimage.

[136] Duerden E.G., Halani S., Ng K., Guo T., Foong J., Glass T.J.A., Chau V., Branson H.M., Sled J.G., Whyte H.E. (2019). White matter injury predicts disrupted functional connectivity and microstructure in very preterm born neonates. Neuroimage Clin..

[137] Lacaille H., Vacher C.M., Penn A.A. (2021). Preterm Birth Alters the Maturation of the GABAergic System in the Human Prefrontal Cortex. Front. Mol. Neurosci..

[138] Vacher C.M., Lacaille H., O’Reilly J.J., Salzbank J., Bakalar D., Sebaoui S., Liere P., Clarkson-Paredes C., Sasaki T., Sathyanesan A. (2021). Placental endocrine function shapes cerebellar development and social behavior. Nat. Neurosci..

[139] Modabbernia A., Mollon J., Boffetta P., Reichenberg A. (2016). Impaired Gas Exchange at Birth and Risk of Intellectual Disability and Autism: A Meta-analysis. J. Autism Dev. Disord..

[140] Modabbernia A., Sandin S., Gross R., Leonard H., Gissler M., Parner E.T., Francis R., Carter K., Bresnahan M., Schendel D. (2019). Apgar score and risk of autism. Eur. J. Epidemiol..

[141] Gardener H., Spiegelman D., Buka S.L. (2011). Perinatal and neonatal risk factors for autism: A comprehensive meta-analysis. Pediatrics.

[142] Giannopoulou I., Pagida M.A., Briana D.D., Panayotacopoulou M.T. (2018). Perinatal hypoxia as a risk factor for psychopathology later in life: The role of dopamine and neurotrophins. Hormones.

[143] Smith T.F., Schmidt-Kastner R., McGeary J.E., Kaczorowski J.A., Knopik V.S. (2016). Pre- and Perinatal Ischemia-Hypoxia, the Ischemia-Hypoxia Response Pathway, and ADHD Risk. Behav. Genet..

[144] Roullet F.I., Lai J.K., Foster J.A. (2013). In utero exposure to valproic acid and autism—A current review of clinical and animal studies. Neurotoxicol. Teratol..

[145] Christensen J., Grønborg T.K., Sørensen M.J., Schendel D., Parner E.T., Pedersen L.H., Vestergaard M. (2013). Prenatal valproate exposure and risk of autism spectrum disorders and childhood autism. JAMA.

[146] Amaral de Lara I.C., de Souza Wagner P.H., Freitas Uchôa Matheus G.T., Eduardo Campos L., de Almeida Souza Miranda C., Cavalcanti Souza M.E., Aquino de Moraes F.C., Alves Kelly F., Rodrigues Fernandes L. (2025). Association of prenatal exposure to antiseizure medication with risk of autism: A systematic review and meta-analysis. Seizure.

[147] Chaliha D., Albrecht M., Vaccarezza M., Takechi R., Lam V., Al-Salami H., Mamo J. (2020). A Systematic Review of the Valproic-Acid-Induced Rodent Model of Autism. Dev. Neurosci..

[148] Watanabe S., Kurotani T., Oga T., Noguchi J., Isoda R., Nakagami A., Sakai K., Nakagaki K., Sumida K., Hoshino K. (2021). Functional and molecular characterization of a non-human primate model of autism spectrum disorder shows similarity with the human disease. Nat. Commun..

[149] Nakagami A., Yasue M., Nakagaki K., Nakamura M., Kawai N., Ichinohe N. (2022). Reduced childhood social attention in autism model marmosets predicts impaired social skills and inflexible behavior in adulthood. Front. Psychiatry.

[150] Noguchi J., Watanabe S., Oga T., Isoda R., Nakagaki K., Sakai K., Sumida K., Hoshino K., Saito K., Miyawaki I. (2024). Altered projection-specific synaptic remodeling and its modification by oxytocin in an idiopathic autism marmoset model. Commun. Biol..

[151] Kataoka S., Takuma K., Hara Y., Maeda Y., Ago Y., Matsuda T. (2013). Autism-like behaviours with transient histone hyperacetylation in mice treated prenatally with valproic acid. Int. J. Neuropsychopharmacol..

[152] Moldrich R.X., Leanage G., She D., Dolan-Evans E., Nelson M., Reza N., Reutens D.C. (2013). Inhibition of histone deacetylase in utero causes sociability deficits in postnatal mice. Behav. Brain Res..

[153] Taleb A., Lin W., Xu X., Zhang G., Zhou Q.G., Naveed M., Meng F., Fukunaga K., Han F. (2021). Emerging mechanisms of valproic acid-induced neurotoxic events in autism and its implications for pharmacological treatment. Biomed. Pharmacother..

[154] Croen L.A., Grether J.K., Yoshida C.K., Odouli R., Hendrick V. (2011). Antidepressant use during pregnancy and childhood autism spectrum disorders. Arch. Gen. Psychiatry.

[155] Hviid A., Melbye M., Pasternak B. (2013). Use of selective serotonin reuptake inhibitors during pregnancy and risk of autism. N. Engl. J. Med..

[156] Kobayashi T., Matsuyama T., Takeuchi M., Ito S. (2016). Autism spectrum disorder and prenatal exposure to selective serotonin reuptake inhibitors: A systematic review and meta-analysis. Reprod. Toxicol..

[157] Bond C.M., Johnson J.C., Chaudhary V., McCarthy E.M., McWhorter M.L., Woehrle N.S. (2020). Perinatal fluoxetine exposure results in social deficits and reduced monoamine oxidase gene expression in mice. Brain Res..

[158] Yu W., Yen Y.C., Lee Y.H., Tan S., Xiao Y., Lokman H., Ting A.K.T., Ganegala H., Kwon T., Ho W.K. (2019). Prenatal selective serotonin reuptake inhibitor (SSRI) exposure induces working memory and social recognition deficits by disrupting inhibitory synaptic networks in male mice. Mol. Brain.

[159] D’Antonio F., Flacco M.E., Valle L.D., Prasad S., Manzoli L., Samara A., Khalil A. (2026). Prenatal paracetamol exposure and child neurodevelopment: A systematic review and meta-analysis. Lancet Obstet. Gynaecol. Women’s Health.

[160] Xu G., Jing J., Bowers K., Liu B., Bao W. (2014). Maternal diabetes and the risk of autism spectrum disorders in the offspring: A systematic review and meta-analysis. J. Autism Dev. Disord..

[161] Rowland J., Wilson C.A. (2021). The association between gestational diabetes and ASD and ADHD: A systematic review and meta-analysis. Sci. Rep..

[162] Rodolaki K., Pergialiotis V., Iakovidou N., Boutsikou T., Iliodromiti Z., Kanaka-Gantenbein C. (2023). The impact of maternal diabetes on the future health and neurodevelopment of the offspring: A review of the evidence. Front. Endocrinol..

[163] Van den Bergh B.R.H., van den Heuvel M.I., Lahti M., Braeken M., de Rooij S.R., Entringer S., Hoyer D., Roseboom T., Räikkönen K., King S. (2020). Prenatal developmental origins of behavior and mental health: The influence of maternal stress in pregnancy. Neurosci. Biobehav. Rev..

[164] Beversdorf D.Q., Manning S.E., Hillier A., Anderson S.L., Nordgren R.E., Walters S.E., Nagaraja H.N., Cooley W.C., Gaelic S.E., Bauman M.L. (2005). Timing of prenatal stressors and autism. J. Autism Dev. Disord..

[165] Manzari N., Matvienko-Sikar K., Baldoni F., O’Keeffe G.W., Khashan A.S. (2019). Prenatal maternal stress and risk of neurodevelopmental disorders in the offspring: A systematic review and meta-analysis. Soc. Psychiatry Psychiatr. Epidemiol..

[166] Kinney D.K., Miller A.M., Crowley D.J., Huang E., Gerber E. (2008). Autism prevalence following prenatal exposure to hurricanes and tropical storms in Louisiana. J. Autism Dev. Disord..

[167] Li J., Vestergaard M., Obel C., Christensen J., Precht D.H., Lu M., Olsen J. (2009). A nationwide study on the risk of autism after prenatal stress exposure to maternal bereavement. Pediatrics.

[168] Class Q.A., Abel K.M., Khashan A.S., Rickert M.E., Dalman C., Larsson H., Hultman C.M., Långström N., Lichtenstein P., D’Onofrio B.M. (2014). Offspring psychopathology following preconception, prenatal and postnatal maternal bereavement stress. Psychol. Med..

[169] Harris A., Seckl J. (2011). Glucocorticoids, prenatal stress and the programming of disease. Horm. Behav..

[170] O’Donnell K.J., Meaney M.J. (2017). Fetal Origins of Mental Health: The Developmental Origins of Health and Disease Hypothesis. Am. J. Psychiatry.

[171] Weisskopf M.G., Kioumourtzoglou M.A., Roberts A.L. (2015). Air Pollution and Autism Spectrum Disorders: Causal or Confounded?. Curr. Environ. Health Rep..

[172] Raz R., Roberts A.L., Lyall K., Hart J.E., Just A.C., Laden F., Weisskopf M.G. (2015). Autism spectrum disorder and particulate matter air pollution before, during, and after pregnancy: A nested case-control analysis within the Nurses’ Health Study II Cohort. Environ. Health Perspect..

[173] Kalkbrenner A.E., Windham G.C., Serre M.L., Akita Y., Wang X., Hoffman K., Thayer B.P., Daniels J.L. (2015). Particulate matter exposure, prenatal and postnatal windows of susceptibility, and autism spectrum disorders. Epidemiology.

[174] Gong T., Almqvist C., Bölte S., Lichtenstein P., Anckarsäter H., Lind T., Lundholm C., Pershagen G. (2014). Exposure to air pollution from traffic and neurodevelopmental disorders in Swedish twins. Twin Res. Hum. Genet..

[175] Guxens M., Ghassabian A., Gong T., Garcia-Esteban R., Porta D., Giorgis-Allemand L., Almqvist C., Aranbarri A., Beelen R., Badaloni C. (2016). Air Pollution Exposure during Pregnancy and Childhood Autistic Traits in Four European Population-Based Cohort Studies: The ESCAPE Project. Environ. Health Perspect..

[176] Chun H., Leung C., Wen S.W., McDonald J., Shin H.H. (2020). Maternal exposure to air pollution and risk of autism in children: A systematic review and meta-analysis. Environ. Pollut..

[177] Rubenstein J.L., Merzenich M.M. (2003). Model of autism: Increased ratio of excitation/inhibition in key neural systems. Genes Brain Behav..

[178] Sohal V.S., Rubenstein J.L.R. (2019). Excitation-inhibition balance as a framework for investigating mechanisms in neuropsychiatric disorders. Mol. Psychiatry.

[179] Liu X., Sun X., Sun C., Zou M., Chen Y., Huang J., Wu L., Chen W.X. (2022). Prevalence of epilepsy in autism spectrum disorders: A systematic review and meta-analysis. Autism.

[180] Hutsler J.J., Zhang H. (2010). Increased dendritic spine densities on cortical projection neurons in autism spectrum disorders. Brain Res..

[181] Blatt G.J., Fatemi S.H. (2011). Alterations in GABAergic biomarkers in the autism brain: Research findings and clinical implications. Anat. Rec..

[182] Fatemi S.H., Reutiman T.J., Folsom T.D., Thuras P.D. (2009). GABA_A_ receptor downregulation in brains of subjects with autism. J. Autism Dev. Disord..

[183] Mori T., Mori K., Fujii E., Toda Y., Miyazaki M., Harada M., Hashimoto T., Kagami S. (2012). Evaluation of the GABAergic nervous system in autistic brain: ^123^I-iomazenil SPECT study. Brain Dev..

[184] Sun L., Grützner C., Bölte S., Wibral M., Tozman T., Schlitt S., Poustka F., Singer W., Freitag C.M., Uhlhaas P.J. (2012). Impaired gamma-band activity during perceptual organization in adults with autism spectrum disorders: Evidence for dysfunctional network activity in frontal-posterior cortices. J. Neurosci..

[185] Buzsáki G., Wang X.J. (2012). Mechanisms of gamma oscillations. Annu. Rev. Neurosci..

[186] Kovarski K., Malvy J., Khanna R.K., Arsène S., Batty M., Latinus M. (2019). Reduced visual evoked potential amplitude in autism spectrum disorder, a variability effect?. Transl. Psychiatry.

[187] Seymour R.A., Rippon G., Gooding-Williams G., Sowman P.F., Kessler K. (2020). Reduced auditory steady state responses in autism spectrum disorder. Mol. Autism.

[188] Ajram L.A., Pereira A.C., Durieux A.M.S., Velthius H.E., Petrinovic M.M., McAlonan G.M. (2019). The contribution of [1H] magnetic resonance spectroscopy to the study of excitation-inhibition in autism. Prog. Neuropsychopharmacol. Biol. Psychiatry.

[189] Thomson A.R., Pasanta D., Arichi T., Puts N.A. (2024). Neurometabolite differences in Autism as assessed with Magnetic Resonance Spectroscopy: A systematic review and meta-analysis. Neurosci. Biobehav. Rev..

[190] Berry-Kravis E., Hagerman R., Visootsak J., Budimirovic D., Kaufmann W.E., Cherubini M., Zarevics P., Walton-Bowen K., Wang P., Bear M.F. (2017). Arbaclofen in fragile X syndrome: Results of phase 3 trials. J. Neurodev. Disord..

[191] Veenstra-VanderWeele J., Cook E.H., King B.H., Zarevics P., Cherubini M., Walton-Bowen K., Bear M.F., Wang P.P., Carpenter R.L. (2017). Arbaclofen in Children and Adolescents with Autism Spectrum Disorder: A Randomized, Controlled, Phase 2 Trial. Neuropsychopharmacology.

[192] Yizhar O., Fenno L.E., Prigge M., Schneider F., Davidson T.J., O’Shea D.J., Sohal V.S., Goshen I., Finkelstein J., Paz J.T. (2011). Neocortical excitation/inhibition balance in information processing and social dysfunction. Nature.

[193] Selimbeyoglu A., Kim C.K., Inoue M., Lee S.Y., Hong A.S.O., Kauvar I., Ramakrishnan C., Fenno L.E., Davidson T.J., Wright M. (2017). Modulation of prefrontal cortex excitation/inhibition balance rescues social behavior in *CNTNAP2*-deficient mice. Sci. Transl. Med..

[194] Wöhr M., Orduz D., Gregory P., Moreno H., Khan U., Vörckel K.J., Wolfer D.P., Welzl H., Gall D., Schiffmann S.N. (2015). Lack of parvalbumin in mice leads to behavioral deficits relevant to all human autism core symptoms and related neural morphofunctional abnormalities. Transl. Psychiatry.

[195] DeLorey T.M., Sahbaie P., Hashemi E., Homanics G.E., Clark J.D. (2008). *Gabrb3* gene deficient mice exhibit impaired social and exploratory behaviors, deficits in non-selective attention and hypoplasia of cerebellar vermal lobules: A potential model of autism spectrum disorder. Behav. Brain Res..

[196] Miyoshi G., Ueta Y., Natsubori A., Hiraga K., Osaki H., Yagasaki Y., Kishi Y., Yanagawa Y., Fishell G., Machold R.P. (2021). *FoxG1* regulates the formation of cortical GABAergic circuit during an early postnatal critical period resulting in autism spectrum disorder-like phenotypes. Nat. Commun..

[197] Gogolla N., Leblanc J.J., Quast K.B., Südhof T.C., Fagiolini M., Hensch T.K. (2009). Common circuit defect of excitatory-inhibitory balance in mouse models of autism. J. Neurodev. Disord..

[198] Neklyudova A., Smirnov K., Rebreikina A., Martynova O., Sysoeva O. (2022). Electrophysiological and Behavioral Evidence for Hyper- and Hyposensitivity in Rare Genetic Syndromes Associated with Autism. Genes.

[199] Lee E., Lee J., Kim E. (2017). Excitation/Inhibition Imbalance in Animal Models of Autism Spectrum Disorders. Biol. Psychiatry.

[200] Berry-Kravis E., Filipink R.A., Frye R.E., Golla S., Morris S.M., Andrews H., Choo T.H., Kaufmann W.E. (2021). Seizures in Fragile X Syndrome: Associations and Longitudinal Analysis of a Large Clinic-Based Cohort. Front. Pediatr..

[201] Nomura T. (2021). Interneuron Dysfunction and Inhibitory Deficits in Autism and Fragile X Syndrome. Cells.

[202] Thibert R.L., Larson A.M., Hsieh D.T., Raby A.R., Thiele E.A. (2013). Neurologic manifestations of Angelman syndrome. Pediatr. Neurol..

[203] Pelc K., Boyd S.G., Cheron G., Dan B. (2008). Epilepsy in Angelman syndrome. Seizure.

[204] Egawa K., Kitagawa K., Inoue K., Takayama M., Takayama C., Saitoh S., Kishino T., Kitagawa M., Fukuda A. (2012). Decreased tonic inhibition in cerebellar granule cells causes motor dysfunction in a mouse model of Angelman syndrome. Sci. Transl. Med..

[205] Ben-Ari Y., Gaiarsa J.L., Tyzio R., Khazipov R. (2007). GABA: A pioneer transmitter that excites immature neurons and generates primitive oscillations. Physiol. Rev..

[206] Pizzamiglio L., Capitano F., Rusina E., Fossati G., Menna E., Léna I., Antonucci F., Mantegazza M. (2025). Neurodevelopmental defects in Dravet syndrome *Scn1a*^+/−^ mice: Targeting GABA-switch rescues behavioral dysfunctions but not seizures and mortality. Neurobiol. Dis..

[207] Cryan J.F., O’Riordan K.J., Cowan C.S.M., Sandhu K.V., Bastiaanssen T.F.S., Boehme M., Codagnone M.G., Cussotto S., Fulling C., Golubeva A.V. (2019). The Microbiota-Gut-Brain Axis. Physiol. Rev..

[208] Desbonnet L., Clarke G., Shanahan F., Dinan T.G., Cryan J.F. (2014). Microbiota is essential for social development in the mouse. Mol. Psychiatry.

[209] Hsiao E.Y., McBride S.W., Hsien S., Sharon G., Hyde E.R., McCue T., Codelli J.A., Chow J., Reisman S.E., Petrosino J.F. (2013). Microbiota modulate behavioral and physiological abnormalities associated with neurodevelopmental disorders. Cell.

[210] Sharon G., Cruz N.J., Kang D.W., Gandal M.J., Wang B., Kim Y.M., Zink E.M., Casey C.P., Taylor B.C., Lane C.J. (2019). Human Gut Microbiota from Autism Spectrum Disorder Promote Behavioral Symptoms in Mice. Cell.

[211] Chaidez V., Hansen R.L., Hertz-Picciotto I. (2014). Gastrointestinal problems in children with autism, developmental delays or typical development. J. Autism Dev. Disord..

[212] Finegold S.M., Dowd S.E., Gontcharova V., Liu C., Henley K.E., Wolcott R.D., Youn E., Summanen P.H., Granpeesheh D., Dixon D. (2010). Pyrosequencing study of fecal microflora of autistic and control children. Anaerobe.

[213] Ho L.K.H., Tong V.J.W., Syn N., Nagarajan N., Tham E.H., Tay S.K., Shorey S., Tambyah P.A., Law E.C.N. (2020). Gut microbiota changes in children with autism spectrum disorder: A systematic review. Gut Pathog..

[214] Kang D.W., Adams J.B., Gregory A.C., Borody T., Chittick L., Fasano A., Khoruts A., Geis E., Maldonado J., McDonough-Means S. (2017). Microbiota Transfer Therapy alters gut ecosystem and improves gastrointestinal and autism symptoms: An open-label study. Microbiome.

[215] Liber A., Więch M. (2025). The Impact of Fecal Microbiota Transplantation on Gastrointestinal and Behavioral Symptoms in Children and Adolescents with Autism Spectrum Disorder: A Systematic Review. Nutrients.

[216] Wan L., Wang H., Liang Y., Zhang X., Yao X., Zhu G., Cai J., Liu G., Liu X., Niu Q. (2024). Effect of oral faecal microbiota transplantation intervention for children with autism spectrum disorder: A randomised, double-blind, placebo-controlled trial. Clin. Transl. Med..

[217] Mitchell K.J., Dahly D.L., Bishop D.V.M. (2026). Conceptual and methodological flaws undermine claims of a link between the gut microbiome and autism. Neuron.

[218] Muller C.L., Anacker A.M.J., Veenstra-VanderWeele J. (2016). The serotonin system in autism spectrum disorder: From biomarker to animal models. Neuroscience.

[219] Israelyan N., Margolis K.G. (2018). Serotonin as a link between the gut-brain-microbiome axis in autism spectrum disorders. Pharmacol. Res..

[220] Wegiel J., Chadman K., London E., Wisniewski T. (2024). Contribution of the serotonergic system to developmental brain abnormalities in autism spectrum disorder. Autism Res..

[221] Schain R.J., Freedman D.X. (1961). Studies on 5-hydroxyindole metabolism in autistic and other mentally retarded children. J. Pediatr..

[222] Hanley H.G., Stahl S.M., Freedman D.X. (1977). Hyperserotonemia and amine metabolites in autistic and retarded children. Arch. Gen. Psychiatry.

[223] Esposito D., Cruciani G., Zaccaro L., Di Carlo E., Spitoni G.F., Manti F., Carducci C., Fiori E., Leuzzi V., Pascucci T. (2024). A Systematic Review on Autism and Hyperserotonemia: State-of-the-Art, Limitations, and Future Directions. Brain Sci..

[224] Terry N., Margolis K.G. (2017). Serotonergic Mechanisms Regulating the GI Tract: Experimental Evidence and Therapeutic Relevance. Handb. Exp. Pharmacol..

[225] Bonnin A., Goeden N., Chen K., Wilson M.L., King J., Shih J.C., Blakely R.D., Deneris E.S., Levitt P. (2011). A transient placental source of serotonin for the fetal forebrain. Nature.

[226] Chandana S.R., Behen M.E., Juhász C., Muzik O., Rothermel R.D., Mangner T.J., Chakraborty P.K., Chugani H.T., Chugani D.C. (2005). Significance of abnormalities in developmental trajectory and asymmetry of cortical serotonin synthesis in autism. Int. J. Dev. Neurosci..

[227] Andersson M., Tangen Ä., Farde L., Bölte S., Halldin C., Borg J., Lundberg J. (2021). Serotonin transporter availability in adults with autism-a positron emission tomography study. Mol. Psychiatry.

[228] Veenstra-VanderWeele J., Muller C.L., Iwamoto H., Sauer J.E., Owens W.A., Shah C.R., Cohen J., Mannangatti P., Jessen T., Thompson B.J. (2012). Autism gene variant causes hyperserotonemia, serotonin receptor hypersensitivity, social impairment and repetitive behavior. Proc. Natl. Acad. Sci. USA.

[229] Toda T., Homma D., Tokuoka H., Hayakawa I., Sugimoto Y., Ichinose H., Kawasaki H. (2013). Birth regulates the initiation of sensory map formation through serotonin signaling. Dev. Cell.

[230] Insel T.R., Hulihan T.J. (1995). A gender-specific mechanism for pair bonding: Oxytocin and partner preference formation in monogamous voles. Behav. Neurosci..

[231] Cho M.M., DeVries A.C., Williams J.R., Carter C.S. (1999). The effects of oxytocin and vasopressin on partner preferences in male and female prairie voles (*Microtus ochrogaster*). Behav. Neurosci..

[232] Kosfeld M., Heinrichs M., Zak P.J., Fischbacher U., Fehr E. (2005). Oxytocin increases trust in humans. Nature.

[233] Modahl C., Green L., Fein D., Morris M., Waterhouse L., Feinstein C., Levin H. (1998). Plasma oxytocin levels in autistic children. Biol. Psychiatry.

[234] Moerkerke M., Peeters M., de Vries L., Daniels N., Steyaert J., Alaerts K., Boets B. (2021). Endogenous Oxytocin Levels in Autism-A Meta-Analysis. Brain Sci..

[235] John S., Jaeggi A.V. (2021). Oxytocin levels tend to be lower in autistic children: A meta-analysis of 31 studies. Autism.

[236] Warrier V., Chee V., Smith P., Chakrabarti B., Baron-Cohen S. (2015). A comprehensive meta-analysis of common genetic variants in autism spectrum conditions. Mol. Autism.

[237] Hollander E., Bartz J., Chaplin W., Phillips A., Sumner J., Soorya L., Anagnostou E., Wasserman S. (2007). Oxytocin increases retention of social cognition in autism. Biol. Psychiatry.

[238] Guastella A.J., Einfeld S.L., Gray K.M., Rinehart N.J., Tonge B.J., Lambert T.J., Hickie I.B. (2010). Intranasal oxytocin improves emotion recognition for youth with autism spectrum disorders. Biol. Psychiatry.

[239] Parker K.J., Oztan O., Libove R.A., Sumiyoshi R.D., Jackson L.P., Karhson D.S., Summers J.E., Hinman K.E., Motonaga K.S., Phillips J.M. (2017). Intranasal oxytocin treatment for social deficits and biomarkers of response in children with autism. Proc. Natl. Acad. Sci. USA.

[240] Yamasue H., Okada T., Munesue T., Kuroda M., Fujioka T., Uno Y., Matsumoto K., Kuwabara H., Mori D., Okamoto Y. (2020). Effect of intranasal oxytocin on the core social symptoms of autism spectrum disorder: A randomized clinical trial. Mol. Psychiatry.

[241] Yamasue H., Kojima M., Kuwabara H., Kuroda M., Matsumoto K., Kanai C., Inada N., Owada K., Ochi K., Ono N. (2022). Effect of a novel nasal oxytocin spray with enhanced bioavailability on autism: A randomized trial. Brain.

[242] Ferguson J.N., Young L.J., Hearn E.F., Matzuk M.M., Insel T.R., Winslow J.T. (2000). Social amnesia in mice lacking the oxytocin gene. Nat. Genet..

[243] Takayanagi Y., Yoshida M., Bielsky I.F., Ross H.E., Kawamata M., Onaka T., Yanagisawa T., Kimura T., Matzuk M.M., Young L.J. (2005). Pervasive social deficits, but normal parturition, in oxytocin receptor-deficient mice. Proc. Natl. Acad. Sci. USA.

[244] Lee H.J., Caldwell H.K., Macbeth A.H., Tolu S.G., Young W.S. (2008). A conditional knockout mouse line of the oxytocin receptor. Endocrinology.

[245] Lin Y.T., Hsieh T.Y., Tsai T.C., Chen C.C., Huang C.C., Hsu K.S. (2018). Conditional Deletion of Hippocampal CA2/CA3a Oxytocin Receptors Impairs the Persistence of Long-Term Social Recognition Memory in Mice. J. Neurosci..

[246] Nishimori K., Sato K., Hidema S., Yoshida M., Mizukami H. (2015). Oxytocin Receptor-Expressing Neurons and Nuclei in the Regulation of Social Behaviors. Interdiscip. Inf. Sci..

[247] Oztan O., Garner J.P., Partap S., Sherr E.H., Hardan A.Y., Farmer C., Thurm A., Swedo S.E., Parker K.J. (2018). Cerebrospinal fluid vasopressin and symptom severity in children with autism. Ann. Neurol..

[248] Yirmiya N., Rosenberg C., Levi S., Salomon S., Shulman C., Nemanov L., Dina C., Ebstein R.P. (2006). Association between the arginine vasopressin 1a receptor (*AVPR1a*) gene and autism in a family-based study: Mediation by socialization skills. Mol. Psychiatry.

[249] Parker K.J., Oztan O., Libove R.A., Mohsin N., Karhson D.S., Sumiyoshi R.D., Summers J.E., Hinman K.E., Motonaga K.S., Phillips J.M. (2019). A randomized placebo-controlled pilot trial shows that intranasal vasopressin improves social deficits in children with autism. Sci. Transl. Med..

[250] Schultz W. (1998). Predictive reward signal of dopamine neurons. J. Neurophysiol..

[251] DiCarlo G.E., Wallace M.T. (2022). Modeling dopamine dysfunction in autism spectrum disorder: From invertebrates to vertebrates. Neurosci. Biobehav. Rev..

[252] Ernst M., Zametkin A.J., Matochik J.A., Pascualvaca D., Cohen R.M. (1997). Low medial prefrontal dopaminergic activity in autistic children. Lancet.

[253] Langen M., Durston S., Staal W.G., Palmen S.J., van Engeland H. (2007). Caudate nucleus is enlarged in high-functioning medication-naive subjects with autism. Biol. Psychiatry.

[254] McAlonan G.M., Cheung V., Cheung C., Suckling J., Lam G.Y., Tai K.S., Yip L., Murphy D.G., Chua S.E. (2005). Mapping the brain in autism. A voxel-based MRI study of volumetric differences and intercorrelations in autism. Brain.

[255] Kosillo P., Bateup H.S. (2021). Dopaminergic Dysregulation in Syndromic Autism Spectrum Disorders: Insights From Genetic Mouse Models. Front. Neural Circuits.

[256] Hamilton P.J., Campbell N.G., Sharma S., Erreger K., Herborg Hansen F., Saunders C., Belovich A.N., Sahai M.A., Cook E.H., Gether U. (2013). De novo mutation in the dopamine transporter gene associates dopamine dysfunction with autism spectrum disorder. Mol. Psychiatry.

[257] Bowton E., Saunders C., Reddy I.A., Campbell N.G., Hamilton P.J., Henry L.K., Coon H., Sakrikar D., Veenstra-VanderWeele J.M., Blakely R.D. (2014). *SLC6A3* coding variant Ala559Val found in two autism probands alters dopamine transporter function and trafficking. Transl. Psychiatry.

[258] DiCarlo G.E., Aguilar J.I., Matthies H.J., Harrison F.E., Bundschuh K.E., West A., Hashemi P., Herborg F., Rickhag M., Chen H. (2019). Autism-linked dopamine transporter mutation alters striatal dopamine neurotransmission and dopamine-dependent behaviors. J. Clin. Investig..

[259] Wei D., Allsop S., Tye K., Piomelli D. (2017). Endocannabinoid Signaling in the Control of Social Behavior. Trends Neurosci..

[260] Zamberletti E., Gabaglio M., Parolaro D. (2017). The Endocannabinoid System and Autism Spectrum Disorders: Insights from Animal Models. Int. J. Mol. Sci..

[261] Navarro D., Gasparyan A., Navarrete F., Torregrosa A.B., Rubio G., Marín-Mayor M., Acosta G.B., Garcia-Gutiérrez M.S., Manzanares J. (2022). Molecular Alterations of the Endocannabinoid System in Psychiatric Disorders. Int. J. Mol. Sci..

[262] Cristino L., Bisogno T., Di Marzo V. (2020). Cannabinoids and the expanded endocannabinoid system in neurological disorders. Nat. Rev. Neurol..

[263] Devane W.A., Dysarz F.A., Johnson M.R., Melvin L.S., Howlett A.C. (1988). Determination and characterization of a cannabinoid receptor in rat brain. Mol. Pharmacol..

[264] Devane W.A., Hanus L., Breuer A., Pertwee R.G., Stevenson L.A., Griffin G., Gibson D., Mandelbaum A., Etinger A., Mechoulam R. (1992). Isolation and structure of a brain constituent that binds to the cannabinoid receptor. Science.

[265] Karhson D.S., Krasinska K.M., Dallaire J.A., Libove R.A., Phillips J.M., Chien A.S., Garner J.P., Hardan A.Y., Parker K.J. (2018). Plasma anandamide concentrations are lower in children with autism spectrum disorder. Mol. Autism.

[266] Aran A., Eylon M., Harel M., Polianski L., Nemirovski A., Tepper S., Schnapp A., Cassuto H., Wattad N., Tam J. (2019). Lower circulating endocannabinoid levels in children with autism spectrum disorder. Mol. Autism.

[267] Purcell A.E., Jeon O.H., Zimmerman A.W., Blue M.E., Pevsner J. (2001). Postmortem brain abnormalities of the glutamate neurotransmitter system in autism. Neurology.

[268] Aran A., Harel M., Cassuto H., Polyansky L., Schnapp A., Wattad N., Shmueli D., Golan D., Castellanos F.X. (2021). Cannabinoid treatment for autism: A proof-of-concept randomized trial. Mol. Autism.

[269] Schmahmann J.D., Guell X., Stoodley C.J., Halko M.A. (2019). The Theory and Neuroscience of Cerebellar Cognition. Annu. Rev. Neurosci..

[270] Limperopoulos C., Bassan H., Gauvreau K., Robertson R.L., Sullivan N.R., Benson C.B., Avery L., Stewart J., Soul J.S., Ringer S.A. (2007). Does cerebellar injury in premature infants contribute to the high prevalence of long-term cognitive, learning, and behavioral disability in survivors?. Pediatrics.

[271] Fatemi S.H., Aldinger K.A., Ashwood P., Bauman M.L., Blaha C.D., Blatt G.J., Chauhan A., Chauhan V., Dager S.R., Dickson P.E. (2012). Consensus paper: Pathological role of the cerebellum in autism. Cerebellum.

[272] Kemper T.L., Bauman M. (1998). Neuropathology of infantile autism. J. Neuropathol. Exp. Neurol..

[273] D’Mello A.M., Crocetti D., Mostofsky S.H., Stoodley C.J. (2015). Cerebellar gray matter and lobular volumes correlate with core autism symptoms. Neuroimage Clin..

[274] Stoodley C.J., D’Mello A.M., Ellegood J., Jakkamsetti V., Liu P., Nebel M.B., Gibson J.M., Kelly E., Meng F., Cano C.A. (2017). Altered cerebellar connectivity in autism and cerebellar-mediated rescue of autism-related behaviors in mice. Nat. Neurosci..

[275] Tsai P.T., Hull C., Chu Y., Greene-Colozzi E., Sadowski A.R., Leech J.M., Steinberg J., Crawley J.N., Regehr W.G., Sahin M. (2012). Autistic-like behaviour and cerebellar dysfunction in Purkinje cell *Tsc1* mutant mice. Nature.

[276] Tsai P.T., Rudolph S., Guo C., Ellegood J., Gibson J.M., Schaeffer S.M., Mogavero J., Lerch J.P., Regehr W., Sahin M. (2018). Sensitive Periods for Cerebellar-Mediated Autistic-like Behaviors. Cell Rep..

[277] Cupolillo D., Hoxha E., Faralli A., De Luca A., Rossi F., Tempia F., Carulli D. (2016). Autistic-Like Traits and Cerebellar Dysfunction in Purkinje Cell *PTEN* Knock-Out Mice. Neuropsychopharmacology.

[278] Peter S., Ten Brinke M.M., Stedehouder J., Reinelt C.M., Wu B., Zhou H., Zhou K., Boele H.J., Kushner S.A., Lee M.G. (2016). Dysfunctional cerebellar Purkinje cells contribute to autism-like behaviour in *Shank2*-deficient mice. Nat. Commun..

[279] Reith R.M., McKenna J., Wu H., Hashmi S.S., Cho S.H., Dash P.K., Gambello M.J. (2013). Loss of *Tsc2* in Purkinje cells is associated with autistic-like behavior in a mouse model of tuberous sclerosis complex. Neurobiol. Dis..

[280] Yamashiro K., Hori K., Lai E.S.K., Aoki R., Shimaoka K., Arimura N., Egusa S.F., Sakamoto A., Abe M., Sakimura K. (2020). *AUTS2* Governs Cerebellar Development, Purkinje Cell Maturation, Motor Function and Social Communication. iScience.

[281] Badura A., Verpeut J.L., Metzger J.W., Pereira T.D., Pisano T.J., Deverett B., Bakshinskaya D.E., Wang S.S. (2018). Normal cognitive and social development require posterior cerebellar activity. eLife.

[282] Kelly E., Meng F., Fujita H., Morgado F., Kazemi Y., Rice L.C., Ren C., Escamilla C.O., Gibson J.M., Sajadi S. (2020). Regulation of autism-relevant behaviors by cerebellar-prefrontal cortical circuits. Nat. Neurosci..

[283] Pisano T.J., Dhanerawala Z.M., Kislin M., Bakshinskaya D., Engel E.A., Hansen E.J., Hoag A.T., Lee J., de Oude N.L., Venkataraju K.U. (2021). Homologous organization of cerebellar pathways to sensory, motor, and associative forebrain. Cell Rep..

[284] Carta I., Chen C.H., Schott A.L., Dorizan S., Khodakhah K. (2019). Cerebellar modulation of the reward circuitry and social behavior. Science.

[285] Fujita H., Kodama T., du Lac S. (2020). Modular output circuits of the fastigial nucleus for diverse motor and nonmotor functions of the cerebellar vermis. eLife.

[286] Hunter D.J. (2005). Gene-environment interactions in human diseases. Nat. Rev. Genet..

[287] Esposito G., Azhari A., Borelli J.L. (2018). Gene × Environment Interaction in Developmental Disorders: Where Do We Stand and What’s Next?. Front. Psychol..

[288] Duncan L.E., Keller M.C. (2011). A critical review of the first 10 years of candidate gene-by-environment interaction research in psychiatry. Am. J. Psychiatry.

[289] Rossignol D.A., Genuis S.J., Frye R.E. (2014). Environmental toxicants and autism spectrum disorders: A systematic review. Transl. Psychiatry.

[290] Keil-Stietz K., Lein P.J. (2023). Gene × environment interactions in autism spectrum disorders. Curr. Top. Dev. Biol..

[291] Volk H.E., Kerin T., Lurmann F., Hertz-Picciotto I., McConnell R., Campbell D.B. (2014). Autism spectrum disorder: Interaction of air pollution with the MET receptor tyrosine kinase gene. Epidemiology.

[292] Campbell D.B., D’Oronzio R., Garbett K., Ebert P.J., Mirnics K., Levitt P., Persico A.M. (2007). Disruption of cerebral cortex MET signaling in autism spectrum disorder. Ann. Neurol..

[293] D’Amelio M., Ricci I., Sacco R., Liu X., D’Agruma L., Muscarella L.A., Guarnieri V., Militerni R., Bravaccio C., Elia M. (2005). Paraoxonase gene variants are associated with autism in North America, but not in Italy: Possible regional specificity in gene-environment interactions. Mol. Psychiatry.

[294] Gaita L., Manzi B., Sacco R., Lintas C., Altieri L., Lombardi F., Pawlowski T.L., Redman M., Craig D.W., Huentelman M.J. (2010). Decreased serum arylesterase activity in autism spectrum disorders. Psychiatry Res..

[295] Paşca S.P., Dronca E., Nemeş B., Kaucsár T., Endreffy E., Iftene F., Benga I., Cornean R., Dronca M. (2010). Paraoxonase 1 activities and polymorphisms in autism spectrum disorders. J. Cell Mol. Med..

[296] Serajee F.J., Nabi R., Zhong H., Huq M. (2004). Polymorphisms in xenobiotic metabolism genes and autism. J. Child. Neurol..

[297] Buyske S., Williams T.A., Mars A.E., Stenroos E.S., Ming S.X., Wang R., Sreenath M., Factura M.F., Reddy C., Lambert G.H. (2006). Analysis of case-parent trios at a locus with a deletion allele: Association of GSTM1 with autism. BMC Genet..

[298] James S.J., Melnyk S., Jernigan S., Cleves M.A., Halsted C.H., Wong D.H., Cutler P., Bock K., Boris M., Bradstreet J.J. (2006). Metabolic endophenotype and related genotypes are associated with oxidative stress in children with autism. Am. J. Med. Genet. B Neuropsychiatr. Genet..

[299] Williams T.A., Mars A.E., Buyske S.G., Stenroos E.S., Wang R., Factura-Santiago M.F., Lambert G.H., Johnson W.G. (2007). Risk of autistic disorder in affected offspring of mothers with a glutathione S-transferase P1 haplotype. Arch. Pediatr. Adolesc. Med..

